# Period teasing, stigma and knowledge: A survey of adolescent boys and girls in Northern Tanzania

**DOI:** 10.1371/journal.pone.0239914

**Published:** 2020-10-28

**Authors:** Anja Benshaul-Tolonen, Sandra Aguilar-Gomez, Naomi Heller Batzer, Rebecca Cai, Elias Charles Nyanza

**Affiliations:** 1 Department of Economics, Barnard College, Columbia University, New York City, NY, United States of America; 2 Columbia University, New York City, NY, United States of America; 3 Department of Environmental and Occupational Health, Catholic University of Health and Allied Sciences, Mwanza, Tanzania; University of the Witwatersrand, SOUTH AFRICA

## Abstract

Emerging evidence suggests that menstruation-related teasing is a common experience among adolescent girls with ramifications on their school participation, yet empirical evidence on the prevalence and determinants of period teasing in schools remain scarce. Menstrual hygiene research and policies almost exclusively focus on girls and women, leading to a dearth of knowledge of male attitudes. We conducted the first quantitative survey of period teasing in schools in sub-Saharan Africa, focusing on 432 male and 524 female students in four co-educational secondary schools in northern Tanzania. Period teasing is prevalent; 13% of girls have experienced period teasing, and more than 80% fear being teased, especially by male classmates. Girls’ fears are associated with insufficient menstrual hygiene management resources and practices. Girls cope by reducing school attendance, participation, and concentration in the classroom during periods. Boys engage in period teasing because they perceive periods as embarrassing, especially visible markers of periods (odor or stains). Social norms, such as peer behavior and home restrictions on menstruating women, are associated with more teasing. Boys believe it is strongly inappropriate for girls to reveal period status or to discuss periods with males, including male teachers. In contrast, boys are well informed about basic biological facts of menstruation (scoring 60% on a knowledge quiz, not statistically different from the girls) and have received information from school curricula and health workers. Lack of suitable menstrual hygiene practices and restrictive social norms is correlated with period teasing, which hinders gender equality in educational opportunities. Providing narrowly bio-medical focused education about menstruation may not be enough to reduce period teasing in contexts with period stigma.

## Introduction

In recent years, an abundance of research and publicity has identified menstruation as an obstacle to gender equality in education, health, and work, particularly in low and middle income countries. Qualitative research has found that a substantial portion of girls’ anxieties about managing their menstruation while at school stems from their fears about boys’ negative reactions [1–5], impacting well-being and school performance. While many studies aim to understand and improve girls’ menstrual hygiene management (MHM), boys’ attitudes and knowledge of the topic remains under-explored and thus limits their inclusion as agents of positive change. As boys grow up, ignorance about the menstrual cycle and women’s reproductive health may result in increased risk of unplanned pregnancy, pregnancy complications, and sexually transmitted infections [6]. Outside of personal relationships, boys and men are deeply influential in determining and perpetuating social attitudes toward menstruation, including prescriptive and proscriptive expectations that limit menstruating women in their daily activities [7, 8]. One acute manifestation of boys’ negative attitudes is period teasing—harassment linked directly to girls’ menstruation. Though early studies [9] highlighted the harms of general and sexual harassment for girls’ educational outcomes, period teasing specifically is a relatively novel and under-explored research topic [5, 10].

In this paper, we perform the first quantitative survey on adolescent boys’ attitudes and knowledge of menstruation in the context of sub-Saharan Africa. In particular, we focus on the prevalence of teasing behavior and its determinants. We contribute to the scant literature on young men’s knowledge and attitudes towards menstruation, previously limited to small studies in the US, Australia, Taiwan and Hong Kong [8, 11–14]), and a few qualitative and mixed-methods studies from low and middle-income countries [15, 16]. In addition, we conduct the first quantitative survey on the prevalence of period teasing in a developing country context, surveying both boys and girls, allowing us to better understand the scope and consequences of the issue. The research builds on socio-medical studies that describe girls’ experiences with menstruation in schools in Tanzania [4, 17, 18], and anthropological work on menstruation in east Africa [7].

### Male attitudes toward menstruation

Despite links with reproductive and public health, little research has focused on boys’ knowledge, learning experiences, and attitudes toward menstruation and the reproductive system. Existing qualitative studies on learning experiences of males in low- and high-income countries report that boys rarely receive comprehensive menstrual health education in school. This results in a piecemeal knowledge acquisition process, misconceptions, and stigma. Surveys of 23 men in the U.S. [11] and 48 men in Australia [14] found that men did not consistently receive information about menstruation in school and that women in their lives treated menstruation as a secret. While some slowly pieced together information and changed their attitudes through dating relationships, others continued to associate periods with “disgust” [11, 14]. Similarly, a larger survey of 287 female and 269 male students in Taiwan revealed more negative attitudes toward menstruation among adolescent men than among women [13]. However, other studies found that men’s lack of knowledge about menstruation is not correlated with negative attitudes; in fact, some men expressed interest in learning more about menstruation [12].

Few studies explore male attitudes toward MHM in developing countries. Only three studies conducted in sub-Saharan Africa were identified [7, 16, 19], in contrast to a large focus on girls’ MHM in this context [4, 17, 18, 20–29]. Similarly, research on adolescent sexual behaviors in developing countries often focuses on girls, with minimal evidence collected on sexual health beliefs and behaviors among teenage boys [30].

In sub-Saharan Africa, the menstrual health research on adolescent males is very limited. Six focus groups with 38 adolescents and five in-depth interviews were conducted in The Gambia [16], and two focus groups of 16 adolescents were conducted in rural Zambia [5]. Similarly to schoolboys in Taiwan, the boys expressed an interest in learning more about menstruation, although mothers did not agree that they should learn about the topic. Focus groups and interviews conducted in five east African countries with key menstruation stakeholders, including some males, highlighted that women were considered unclean during menstruation and were not allowed to participate in public gatherings [7]. In addition, a recent multifaceted intervention in Uganda explores menstrual knowledge, myth and perception test among 218 boys [19].

A few studies have focused on male attitudes and knowledge of MHM in India. The research most similar to ours is a study of 85 adolescent boys across 8 schools in three states in India [15], which found a similar piecemeal pattern of knowledge acquisition, and a variety of attitudes toward menstruation ranging from sympathy among the majority to disgust in the minority [15]. A non-randomized sensitisation program rolled out to men and adolescent boys in Uttar Pradesh, India led to increased discussion of menstrual hygiene among males [31]. In a survey of one male teacher and twelve female teachers in Mumbai, teachers reported that resistance to boys’ menstrual health education comes from parents, some teachers, and female classmates [32]. Revealingly, the study intended to survey equal proportions of male and female teachers, but the majority of male teachers refused to be interviewed out of apparent gendered discomfort with the topic of menstruation [32].

In formulating policy interventions in the future, it will be necessary to understand how males—as classmates, family members, and teachers—think about menstruation and what causes these beliefs. Empirical evidence to date mentions, but rarely tests, the determinants of boys’ knowledge and how knowledge may shape attitudes. Studies have found that age, education, urban/rural setting, and community attitudes may be associated with knowledge and restrictiveness of beliefs. For instance, while married men in rural Rajasthan, India believe that women can pollute everything they touch during menstruation [33], Indian boys aged 13-17 believe that it would be beneficial to reduce stigma so that girls could be more open about their menstrual status [15].

Exposure to education that contradicts otherwise deeply rooted norms likely mediates these generational differences. A study among Hong Kong undergraduates found that students in non-health-related programs exhibited higher levels of belief in traditional limitations on menstruating women [8]. To our knowledge, only two studies using the same data in Uttar Pradesh have performed quantitative analysis on the determinants and relationship between individuals’ knowledge and attitudes on a large sample [6, 34], although these two studies examine adult men’s general knowledge of women’s reproductive health and include menstruation only as a small component.

## Methods

We use original data obtained from written surveys of 432 boys and 524 girls across four co-educational schools in two districts in northern Tanzania. The survey tested knowledge of the biological facts of menstruation, and inquired about teasing behavior, norms, and stigma.

The paper is organized into the following sections, according to the aims of the paper. We first compare boys’ and girls’ knowledge, sources of information, and attitudes within the same secondary schools. Then we proceed to the main focus of the paper: the prevalence of period teasing behavior and its determinants. We compare boys’ and girls’ experiences of period teasing to estimate the proportion of boys who have ever teased and proportion of girls who have ever been teased. Further, we analyze boys’ reasons for teasing, deepening our understanding of underlying dynamics that perpetuate girls’ fears and shame around menstruation, especially in school settings. Further, we test for individual correlates that increase the likelihood that boys engage in period teasing. Finally, we innovate by testing the link between boys’ attitudes and the norms and expectations around menstruation in their homes.

### Sampling

The sampled students were enrolled at four co-educational secondary schools in Sengerema District (Mwanza Region) and Geita District (Geita Region), Tanzania. Systematic sampling was done using class register, where every third student was selected to participate in the study provided they were present at the school. In total, 432 male and 524 female students participated in the study. The schools were rural, although one schools was peri-urban. One school had a girls-only hostel, and the other three schools were day-schools only. While the selection of the four schools is not representative of the schools in the regions, the in-school sample is representative as we used a random sample of all present students in the target secondary school grades (1 through 3). We cannot determine how the selected schools compare to the schools in the region because of lack of data.

Data collection was undertaken in 2019 with a mixed gender research team. All research staff were present in the classrooms during the surveying, providing a mixed-gender research group. Classroom teachers were involved to help hand out and collect the surveys and to keep order in the classrooms.

To avoid congestion of students in classrooms, special classrooms were assigned for the study, up to six rooms per school. Boys and girls were separated. Surveys were collected using pen and paper, as we determined this would allow the students the maximum amount of privacy and honesty in reporting, compared to an enumerator-led survey.

**Fig 1:**
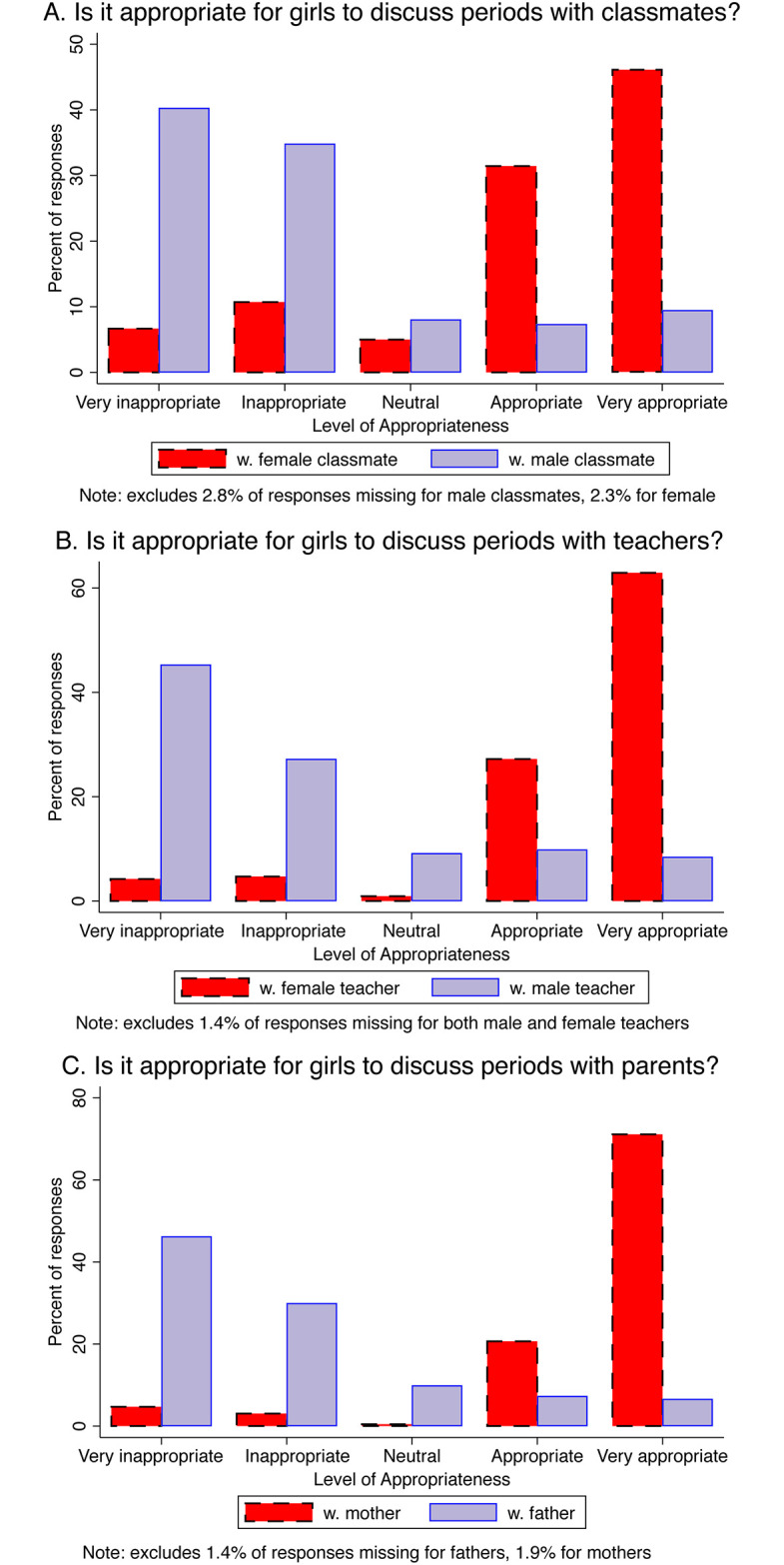
Boys’ attitudes toward propriety of girls discussing periods. Notes: The sample consists of 432 boys excluding the percentage of missing responses reported by figure.

The survey contained a knowledge assessment section (listed in Fig 1), and questions about menstruation-related experiences, attitudes, information sources, and teasing behavior. The original Kiswahili questionnaires with English translations are included in the Supplementary Information. The questionnaires were validated by research assistants from a local university The survey was additionally tested on a US population prior. No further validation was done as the data collection was part of a pilot initiative, with potential implications for accuracy and scope. The survey contained 10 factual questions about menstruation, including menstruation frequency, duration, age of onset, connections with fertility, and physical symptoms. A list of all knowledge questions is in S1 Appendix. We constructed an index to measure the proportion of the 10 questions each boy answered correctly, as a measurement of his overall knowledge of menstruation. The possible values of knowledge score index range (0-1).

### Statistical analysis

All statistical analysis was performed with Stata v.15.1. Statistical significance of gender differences between female and male student samples is calculated with a two-sample t-test for unequal variance between groups, using Welch’s approximation. In Table 4 we test determinants of period teasing and negative attitudes toward periods with OLS regression, controlling for age, grade, and school fixed effects. Regression analysis performed with heteroskedasticity-robust (Huber-White) standard errors.

### Ethical considerations

Research permits for the study were received at the Bugando Medical Centre and Catholic University of Health and Allied Science Joint Ethics and Review Committee (CREC/360/2019), and National Institute for Medical Research (NIMR) (Ref: MR/53/100/596) in Tanzania. IRB was approved at Barnard College, Columbia University (Ref: 1819-1110-010) in the USA. Permission to conduct the research were obtained from the Tanzania Commission for Research and Technology (COSTECH) and at Geita District Council (Ref: GDC/E.10/1/VOL.2/267).

The data was collected within four selected schools in Sengerema and Geita Districts. Permission to collect data in secondary schools in the respective districts was obtained from the district medical officers of health and district secondary school education officers.

An assent form in Kiswahili (the primary language of most of the population of Tanzania) that explained the purpose, risks, significance, and the right to either participate or withdraw from the study was obtained prior collection of data. All participating students provided a written assent. Respective parents were informed on the research activities through their school management committees. In addition, each school’s headmasters or headmistresses gave a written consent for their school participation in the study.

## Results

Baseline summary statistics are presented in Table 1. On average, boys are 15.92 years-old and girls are 15.21 years-old, and the age range is quite wide. This is plausibly due to delayed school start and high level of grade retake common in the area. All students are in grades 1 to 3 of secondary school, which follows six years of primary school in the Tanzanian school system.

**Table 1:** Baseline demographics and experience with menstruation.

	(1) Boys	(2)	(3)	(4)	(5)	(6) Girls	(7)	(8)	(9)	(10)	(11) Diff.
**Panel A: continuous variables**	Mean	SD	Min	Max	N	Mean	SD	Min	Max	N	
*Demographics*											
Age (years)	15.92	1.58	13	22	382	15.21	1.52	10	22	458	-0.71***
Grade (secondary school 1-3)	1.76	0.75	1	3	423	1.71	0.72	1	3	503	-0.05
Duration of residence (years)	9.46	5.53	0	19	402	7.28	5.78	0	19	447	-2.18***
Monthly expenditure (1000s THS)	21.10	30.64	0	300	334	6.67	17.34	0	300	361	-14.43***
*Menstrual knowledge and peer teasing*											
Menstrual knowledge score index	0.60	0.19	0	1	432	0.61	0.16	0	1	524	0.01
No. male friends observed teasing	0.37	0.99	0	5	419	.	.	.	.	.	.
**Panel B: binary variables (no = 0, yes = 1)**	Share Yes		[95% CI]		N	Share Yes		[95% CI]	N		
*Information on menstruation*											
Someone has told me about periods	0.79		0.75	0.83	417	0.90		0.87	0.93	448	0.11***
*Received information from*:											
school curricula	0.63		0.57	0.68	355	0.76		0.71	0.80	350	0.13***
health worker	0.59		0.54	0.65	355	0.80		0.75	0.83	396	0.20***
informational pamphlet	0.30		0.24	0.36	255	0.45		0.39	0.51	297	0.15***
Internet	0.26		0.21	0.31	325	0.40		0.35	0.46	323	0.14***
*Boys’ attitudes*: Agree that girls…											
should get married after menarche	0.05		0.03	0.07	327	.		.	.	.	.
are ready for sex after menarche	0.32		0.27	0.37	320	.		.	.	.	.
*Boys’ period teasing*											
Has teased girls	0.18		0.15	0.22	403	.		.	.	.	.
*Girls’ period teasing*											
Ever been teased about periods	.		.	.	.	0.13		0.10	0.17	486	.
Afraid of teasing: leaking	.		.	.	.	0.80		0.76	0.83	441	.
Afraid of teasing: odor	.		.	.	.	0.87		0.84	0.90	435	.
Number of participants	432					524					

Notes: Welch test for unequal variance between groups in column 11.

* *p* < 0.10,

** *p* < 0.05,

*** *p* < 0.01.

Knowledge score index is the proportion of 10 questions about biological facts of menstruation answered correctly by the respondent. Given the skew distribution of monthly expenditure, we checked robustness of the significant gender difference by performing a t-test on natural log of monthly expenditure. The result still holds (*p* < 0.01).

### Sources of information

Boys receive information about girls’ periods from multiple sources, including direct communication (Table 1). Of the subset of boys who reported they had received information, 63% received information from school curricula, 59% from a health worker, 30% from informational pamphlets like ads and posters, and 26% from the Internet. Girls were more likely than boys to receive interpersonal instruction on menstruation (90% of girls compared to 79% of boys. The most common sources of information for girls were health workers (80%) and school curricula (76%). In sum, boys reported receiving slightly less interpersonal and formal education about periods than girls, although the majority of boys and girls receive information from multiple sources.

Boys consider it inappropriate for girls to discuss periods with male classmates, male teachers, or fathers, potentially hindering the informal flow of information on menstruation. In two separate questions, the survey prompted boys to answer whether it is appropriate for girls to discuss periods with female classmates, and with male classmates (Fig 1A). Fig 1B reports the distribution for the same questions regarding female versus male teachers, and Fig 1C for mothers versus fathers. In all three cases, boys displayed a very clear pattern: they overwhelmingly support girls speaking to female classmates, female teachers, and mothers, but heavily disapprove of girls speaking to male counterparts.

### Period teasing and classroom participation

**Fig 2:**
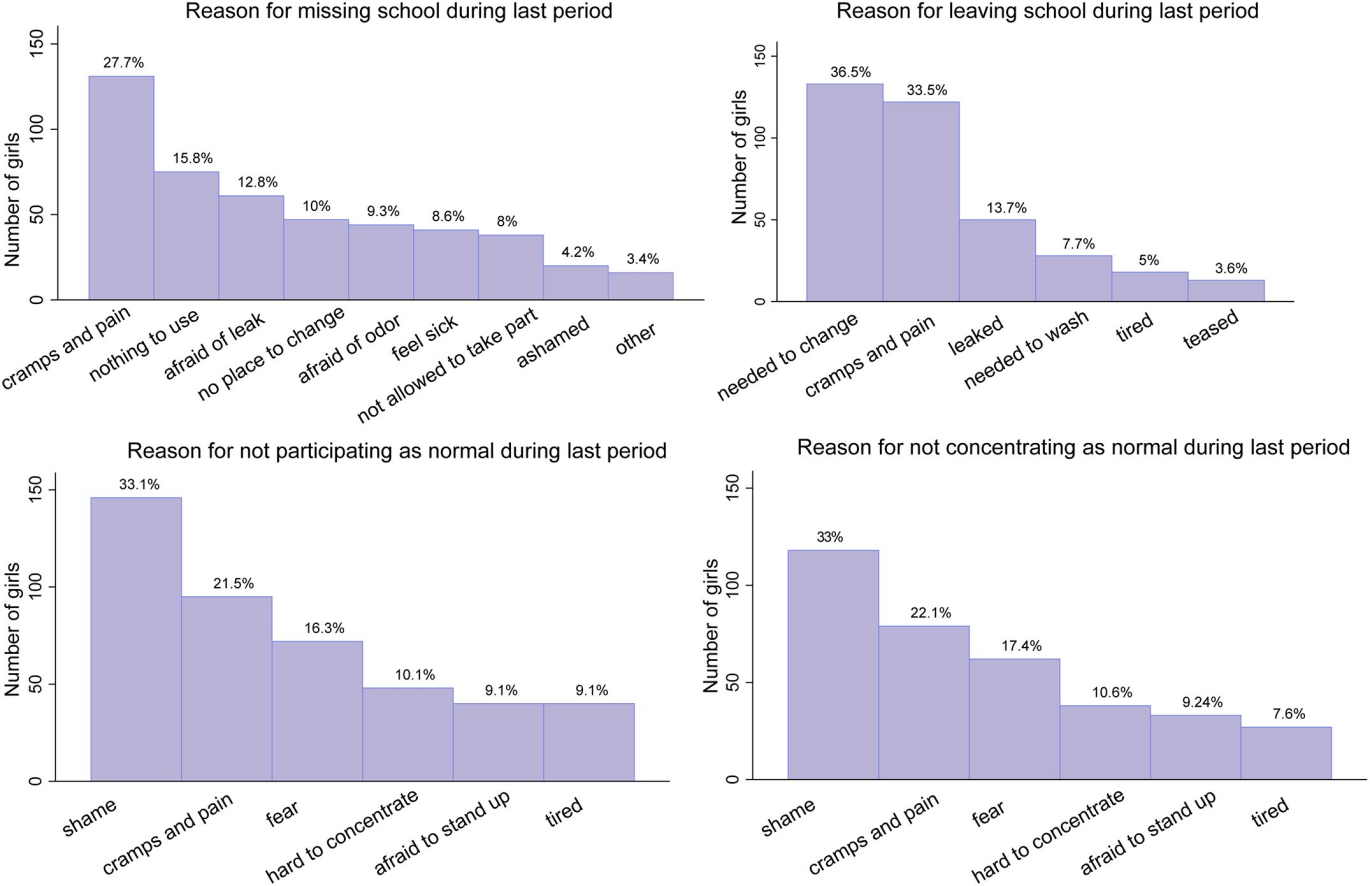
Girls’ school attendance, participation and concentration during last period.

Teasing and fear of teasing is among the girls: 13% of girls report that they have ever experienced period teasing, 87% fear being teased because of menstrual odor, and 80% fear being teased because of leaking (Table 1). 47% of girls left school early during their last period, 31% did not participate in class as much as normal, and 33% concentrated less while in school. Fear and shame, alongside cramps and pain, are commonly reported reasons for absenteeism or lower participation and concentration in the classroom (Fig 2). Of the girls who left school early, roughly 4% did so because they experienced period teasing. One reason why girls may participate less in the classroom during their period is because students are required to stand up while answering questions, potentially revealing that they are menstruating. 9% of girls report being afraid to stand up, and an additional 33% participated less because of shame and 16% because of fear.

**Fig 3:**
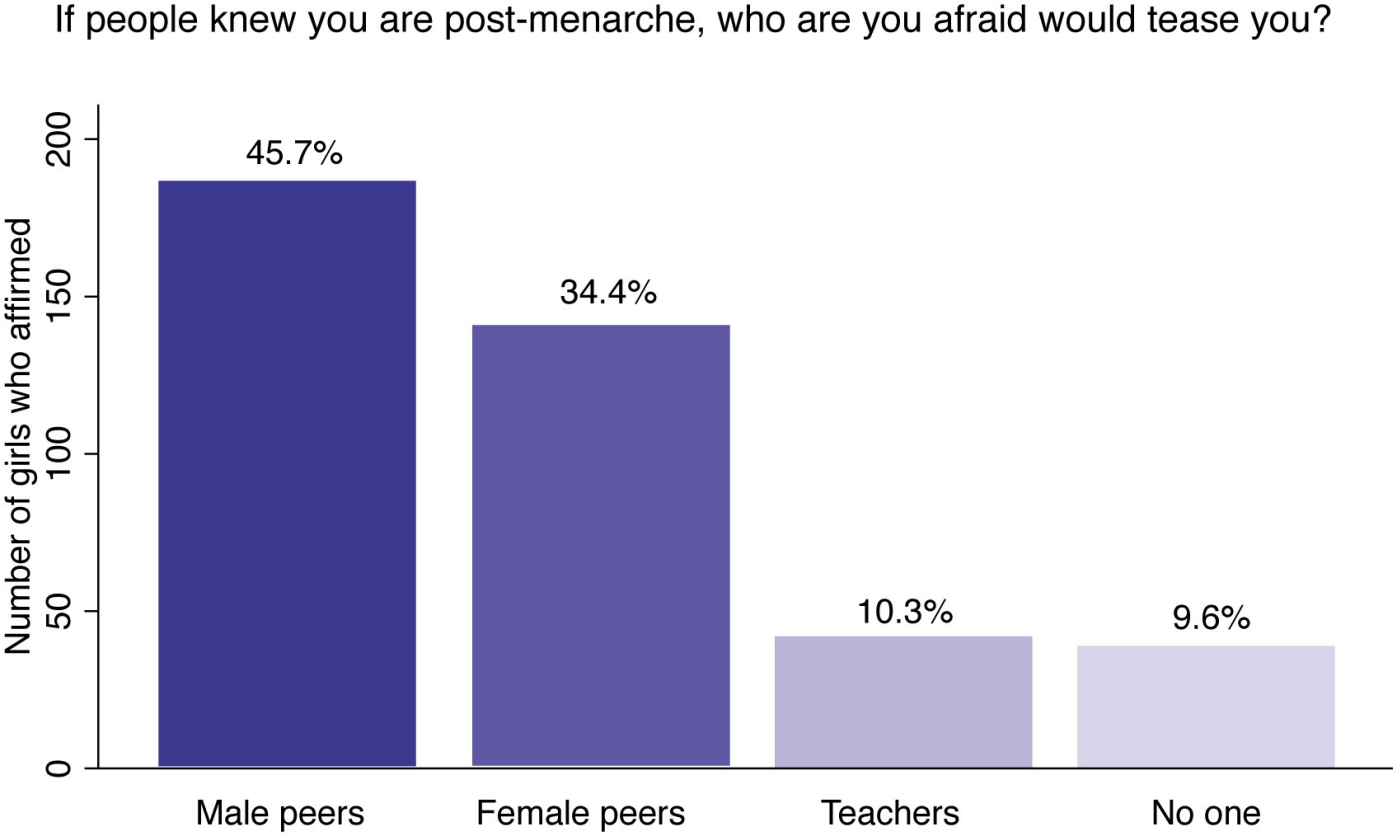
Girls’ fear of being teased based on identity of perpetrator.

The survey prompted the girls to reveal the identity of the people they fear would tease them, if people knew they are post-menarche. The plurality of girls (46%) report male peers, followed by female peers (34%), teachers (10%) and no one (10%) (Fig 3). This confirms that male peers are the most commonly feared perpetrator of period teasing in co-educational schools, although the results may indicate that female peers and teachers play a significant role in teasing as well.

**Fig 4:**
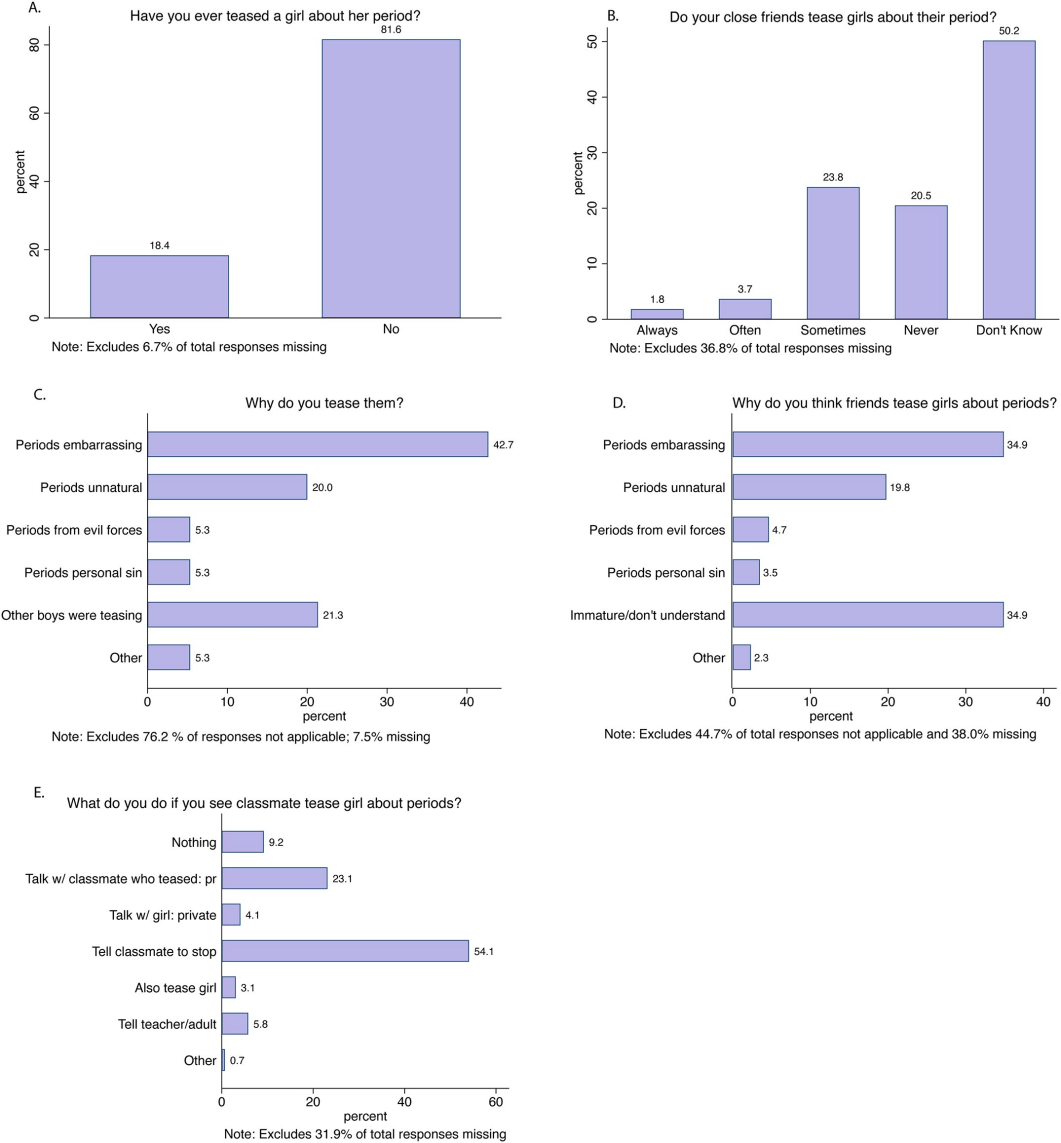
Boys’ and male friends’ reported teasing behavior. Notes: The sample consists of 432 boys excluding the percentage of missing responses reported by figure. Answer to question listed in Fig C is conditional to answering “yes” to question reported in Fig A.

**Fig 5:**
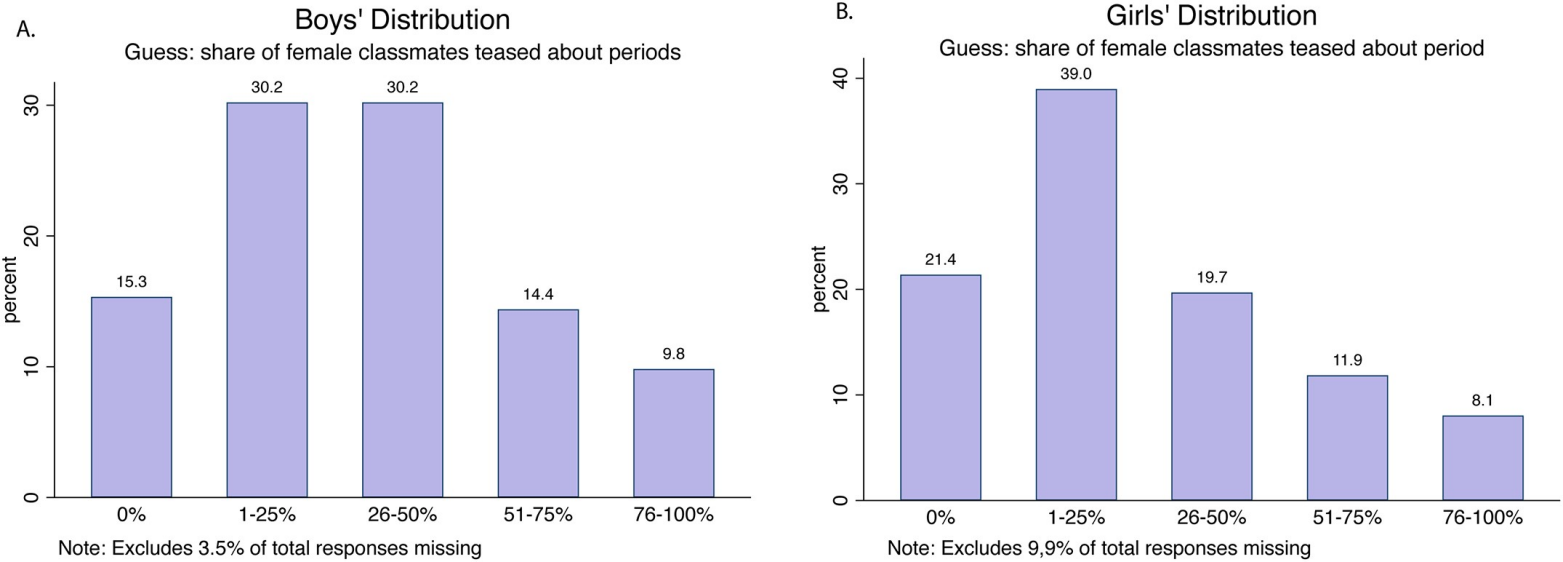
Boys’ and girls’ guesses on teasing prevalence.

In light of this fear, 18% of boys report that they have ever personally teased a girl about her period (Fig 4A), and 29% have observed their close friends teasing (Fig 4B). Further, girls and boys were most likely to guess that 1-25% of their female peers had been teased, showing an awareness of teasing and its relative prevalence (see Fig 5). Interestingly, boys were equally likely to guess that a higher-than-actual proportion (26-50%) of their female classmates had been teased (Fig 5A).

### Reasons for period teasing

Boys tease because “periods are embarrassing” (43% of boys who had teased), “other boys were teasing” (21%), and “periods are unnatural” (20%) (Fig 4C). Boys’ guesses for why their friends teased girls ranged from “periods are embarrassing” (35%), “boys are immature and don’t understand” (35%), and “periods are unnatural” (20%) (Fig 4D). The high share of boys reporting that they tease because other boys were teasing (21%) points to significant peer pressure (Fig 4D), in line with [30], who finds evidence for strong peer pressure around sexual behavior among adolescent boys in Tanzania.

The boys were further probed to explain what specific circumstances caused them, or their friends, to tease girls (Table 2). Boys reported that teasing happens when girls have an odor (38% for self, 40% for friends), or when girls’ clothes are stained with blood (27% and 20%, respectively), both of which may occur when girls cannot practice proper menstrual hygiene management due to lack of resources or infrastructure. Our concurrent survey of girls reveals that girls fear teasing for the same reasons: an overwhelming proportion of girls feared teasing for leaking menstrual blood at school (80%) or feared teasing for menstrual odor (87%), even though many reported they had never been teased (Table 1).

**Table 2:** When I/classmates tease girls about periods.

Reasons for teasing	Respondent teased		Respondent’s friend(s) teased	
	Freq (Percentage)	N	Freq (Percentage)	N
When they smell bad	28 (38.4)	74	35 (39.7)	88
When their clothes get blood stains	20 (27.0)	74	18 (20.4)	88
When I/they find pads or other products	25 (33.8)	74	9 (10.2)	88
When girls don’t participate in sports or class	12 (16.2)	74	14 (15.9)	88
When I/they think that girl has period	14 (18.9)	74	11 (12.5)	88
Other reason	2 (2.7)	74	1 (1.1)	88

### Accuracy of beliefs and attitudes toward menstruation

Lack of knowledge about menstruation could potentially lead to more teasing behavior, especially because 20% of boys reported “periods are unnatural” as a reason for period teasing. We explore this hypothesis by first quantifying boys’ knowledge of the menstrual cycle.

**Fig 6:**
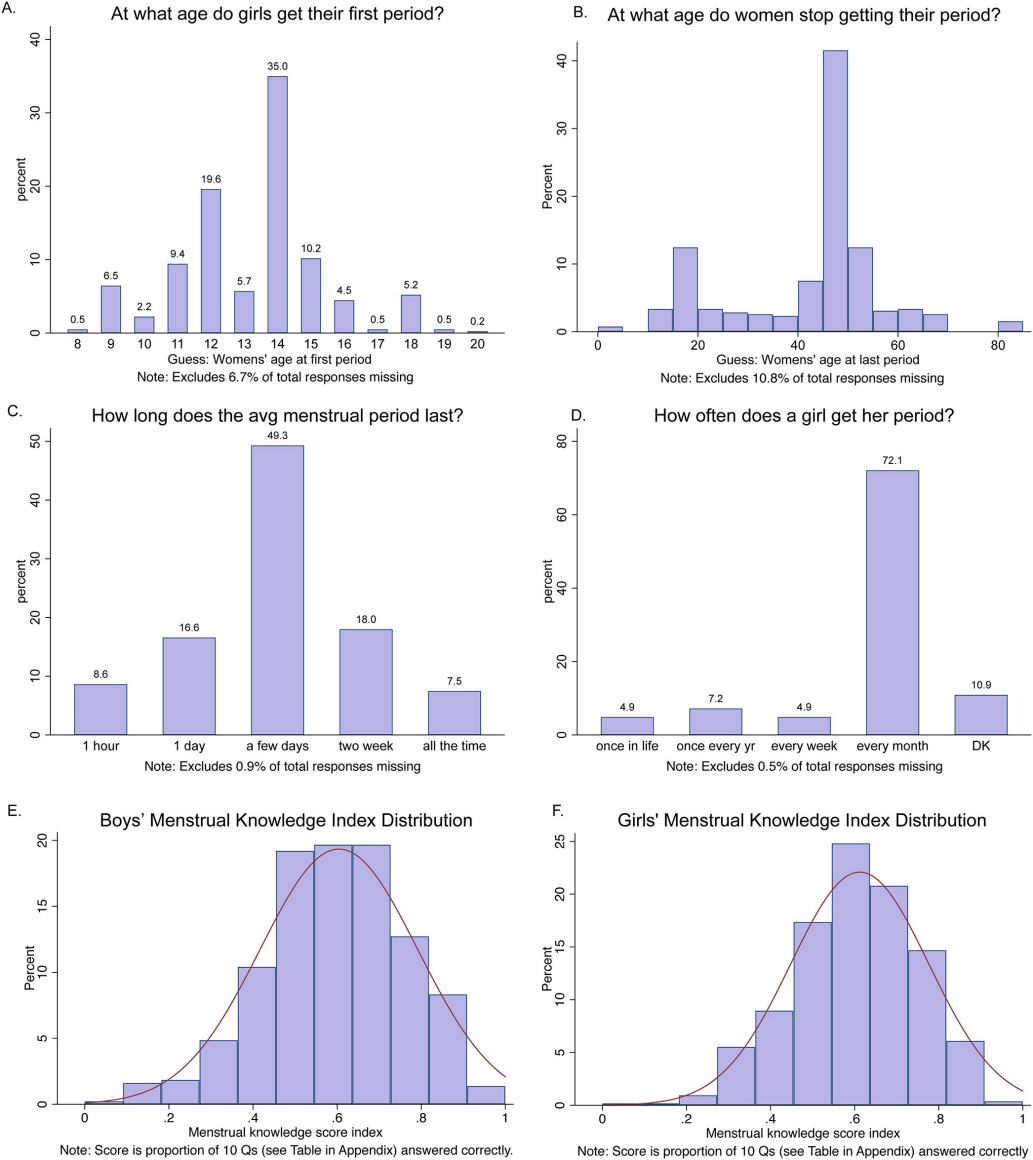
Boys: (A) Age at Menarche and (B) Menopause, and (C-D) Length of Menstrual Cycle, and (E-F) Boys’ and Girls’ Menstrual Knowledge Index.

Most boys (72%) were aware that a girl gets her period approximately every month (Fig 6D). One caveat with this finding is that in Kiswahili, the word for month (*mwezi*) is the same as the informal word for menstruation. A lower proportion of boys (49%) answered correctly that a woman’s period on average lasts a few days (Fig 6C).

We estimated girls’ average age of menarche within a 95% confidence interval of (14.2 ± 2.2 years), aligned with previous research (14.3±2.2 yrs) in two other regions of Tanzania (Dar Es Salaam and Mafinga) [35]. There are no representative estimates for age of menopause in our study’s two regions, so we define correct age range for menopause based study of 150 women in rural Singida, Tanzania (50.1 ± 7.6 yrs) [36]. Boys were more precise in guessing age of menarche. The mean of their guess (13.28) is correct and the distribution is fairly tight (SD = 2.17) (Fig 6A). By contrast, their guesses for the age of menopause were widely varied. The mean (40.43) is too low, and the distribution of guesses is very wide (SD = 14.54) (Fig 6B). As a comparison to test boys’ relative accuracy of beliefs, the girls on average guessed a medically accurate age of menarche (12.37), although this is below the average in the study schools. The girls’ guess for age of menopause is closer to the correct age (46.5) and has a tighter distribution (SD = 10.21), perhaps due to differences in school health curriculum or closer observation of female family members.

Boys averaged 60% correct on the 10-question knowledge test. Fig 6E and 6F compare the distributions of the boys’ and girls’ knowledge score indices. Overall, the girls’ scores distribution is marginally higher than the boys, but the difference is not statistically significant at the 10% level (t-stat = -1.18) using a two-sample Welch t-test for unequal variance in score among girls and boys.

### Period stigma at school and in the home

Boys report that their school and home environments generally stigmatize menstruation as something that should be hidden (Table 3). 79% of boys answered that girls at school would be ashamed to reveal their menstruation to male classmates, although only 27% of boys stated that they knew when female classmates were menstruating. Boys weakly agree that women and girls in their homes should be ashamed to reveal their menstruation (mean of 3.35 where 1 is strongly disagree and 5 is strongly agree).

**Table 3:** Boys’ attitudes toward menstruation.

**Panel A: continuous variables**	Mean	SD	Min	Max	N
Appropriate for girls to talk about periods in public? (1-5)	2.173	1.317	1	5	423
Women in my house ashamed to reveal menstruation (1-5)	3.355	1.564	1	5	425
**Panel B: categorical variables**	Mean		[95% CI]		N
Do you feel safe in school latrines? (Y/N)	0.27		0.23	0.31	423
Do you know when your classmates have their periods? (Y/N)	0.27		0.23	0.31	428
Do you think girls would be ashamed if you knew? (Y/N)	0.78		0.74	0.82	423
Do you feel periods should be hidden at school? (Y/N)	0.53		0.48	0.58	426
Would you like to have someone you can ask about periods? (Y/N)	0.84		0.80	0.87	402
Menst’ing women in my house restricted from activities (Y/N)	0.66		0.61	0.71	371
If YES, restricted from…					
…touching water sources/animals	0.17		0.13	0.21	432
…cooking	0.29		0.25	0.33	432
…washing dishes	0.16		0.13	0.20	432
…public gatherings	0.17		0.14	0.21	432
…sleeping in normal spot	0.06		0.04	0.08	432
…using normal toilet/latrine	0.08		0.06	0.11	432
…other	0.06		0.04	0.09	432

These positive and normative proscriptions are accompanied with physical restrictions in the home. 66% of boys reported that menstruating women and girls in their homes are restricted from daily activities, most commonly cooking (50% of restrictionist households). Touching water sources or animals, washing dishes, and attending public gatherings are restricted in roughly 30% of restrictionist households.

Finally, we investigate whether boys identify menstruation with sexual maturity and with marriageability by asking: 1)“Do you agree girls should get married as soon as they get their period?”; and 2)“Do you agree girls are old enough to have sex after they get their first period?”. We find that while they do not associate menstruation with marriageability (5% agree or strongly agree), boys are more likely to associate menstruation with sexual maturity (32% agree or strongly agree). The implication is that almost 1 in 3 boys believe that girls are sexually mature after they have started menstruation. This portion of boys ascribe sexual maturity to relatively young girls, as our concurrent survey from girls in the same schools found the actual average age of menarche was 14.2 years.

Despite the prevalence of shame and proscription surrounding menstruation in boys’ lives, there are also reasons to believe boys have a role in promoting healthier MHM. Boys are curious to learn more, as 84% answered that they would like to have someone they can ask about periods (Table 3, in line with results from focus group studies of adolescents in Taiwan [12] and in India [15].

### Determinants of period teasing

In this section, we test whether menstrual knowledge, home environment, and peer behavior are significantly associated with boys’ attitudes and behaviors toward periods. We measure negative behaviors with a binary indicator for whether a boy has ever teased girls about periods (Columns 1-4) (Table 4). In the same table, we measure negative attitudes with two indicators for whether a boy believes it is appropriate for girls to publicly discuss periods (Column 5), and whether periods should be hidden in school (Column 6).

**Table 4:** Boys’ attitudes toward periods and period-related teasing based on menstrual knowledge, home environment, and friends’ period teasing.

	Reported period-teasing				Negative attitudes	
	Have teased girls about periods *bin(0/1)*				Appropriate if girls publicly discuss periods *bin(0/1)*	Periods should be hidden in school *bin(0/1)*
	(1)	(2)	(3)	(4)	(5)	(6)
*Mean*:	0.184	0.184	0.184	0.184	0.206	0.531
Menstrual knowledge score index *continuous [0, 1]*	0.0152	-0.0975	0.0387	0.0438	-0.00429	0.160
	(0.13)	(-0.86)	(0.33)	(0.38)	(-0.03)	(0.97)
Home restricts menstruating women *binary (0/1)*	0.0974**		0.100**	0.0979**	0.0459	-0.00543
	(2.25)		(2.38)	(2.31)	(0.98)	(-0.09)
Number of home activities restricted during menstruation *count [0, 6]*		0.0502***		0.0463**		
		(2.45)				
Report any male friends have teased *binary (0/1)*			0.212***		-0.0156	0.00144
			(3.10)		(-0.26)	(0.02)
Number of male friends who have teased *count [0, 5]*				0.0985***		
				(3.35)		
Observations	314	353	310	314	328	325

Note: All columns report OLS coefficients. *t* statistics in parentheses.

* *p* < 0.10,

** *p* < 0.05,

*** *p* < 0.01.

Robust standard errors (Huber-White estimators). Dependent variables: The outcome in columns 1-4 is a binary variable that equals 1 if a boy reports that he has ever teased girls about periods. Outcome in column 5 is a binary indicator equalling 1 if a boy agrees that is appropriate/very appropriate for girls to discuss periods. Column 6 is a binary indicator equalling 1 if a boy agrees that periods are something to be hidden in school. Controls (not reported) are age and grade. School fixed effects included.

One might expect that boys who perform poorly on a 10-question test about biological facts of menstruation are more likely to stigmatize periods and engage in period teasing, if, for example, such boys were more likely to believe that periods are unnatural or embarrassing. However, we do not find a statistically significant correlation between boys’ menstrual knowledge score (the proportion of correct answers on the 10-question test, see S1 Appendix), and likelihood of period teasing or negative attitudes (Table 4 Row 1). The menstrual knowledge score is not correlated with reported teasing or negative attitudes, across the six different model specifications.

However, boys who report restrictions on menstruation in their homes are more likely to engage in period teasing. Boys who report that their home restricts at least one behavior are 9.7 percentage points (*p* < 0.05) more likely to report they have teased (Table 4, Column 1). Relative to the sample mean, this represents a 53% increase in likelihood of teasing. This result is robust to an alternative specification using the number of restricted activities as the determinant; an additional home restriction is associated with a highly significant (*p* < 0.01) 5.0 percentage point increase in likelihood of reporting period teasing (Table 4, Column 2). The robustness of this result is further explored in Table 1, which examines each of seven restricted activities separately (S1 Appendix).

Further, there is significant evidence of peer effects on the likelihood of period teasing. Controlling for boys’ menstrual knowledge and home restrictions, boys who report that any of their five closest male friends have teased girls over periods are 21.2 percentage points (*p* < 0.01) more likely to report personally engaging in teasing (Table 4, Column 3), with alternative specification in Column 4. Reporting one additional friend who has teased is associated with 9.9 percentage point (*p* < 0.01) increase in likelihood of self-reported period teasing.

Neither menstrual knowledge score, nor home restrictions, nor peers’ teasing behavior are significantly correlated with beliefs that periods should not be publicly discussed or revealed at school (Table 4, Columns 5 and 6).

## Discussion

This study marks the largest sample to-date measuring adolescent boys’ knowledge, experience, and attitudes surrounding menstruation, and the first study to collect information on boys’ period teasing behavior. In addition, the study provides the first quantitative measures of the prevalence of period teasing and girls’ anxieties around teasing in a developing country. Finally, we provide insight into individual determinants of teasing behavior among adolescent boys.

### Key results and implications

A non-trivial share of the girls (13%) report having experienced period teasing, and fear of period teasing is almost universal (over 80%). The majority of previous qualitative studies that survey girls portray teasing as a common phenomenon and a major source of anxiety for girls [3, 5]—we confirm this finding. The only other quantitative study to date, which was a non-representative poll of 1,000 U.K. girls aged 14-21, revealed that one in five girls have experienced teasing or bullying surrounding their periods [37].

Boys report personally engaging in period teasing (18%), and observing their close male friends teasing (29%). The true prevalence of period teasing may be higher because of a reluctance to report it. This may be the case both among perpetrators and victims. One indication of potential under-reporting is the large share (30%) of boys who chose not to answer whether they had witnessed their friends tease. On the other hand, very few boys (7%) refused to answer whether they themselves had teased. The high rate of attrition for questions about friends indicates that boys may have experienced discomfort when asked to reveal if their friends had committed “bad” behaviors despite the fact that they did not have to name the perpetrators. This is in line with previous findings from other contexts: boys in India denied that any of their male peers had ever teased menstruating girls, though qualitative evidence indicated otherwise [15].

Boys were prompted to tease by the perception that periods are embarrassing, and incidences wherein girls reveal bad odor and blood stains. This supports qualitative evidence, including studies in neighboring countries, that much of girls’ anxieties surrounding periods comes from the possibility of revealing their period through blood, odor, or discarded sanitary products, and the social risks associated with these type of incidences [2, 3, 20, 38]. Our results strongly align with focus group discussions in rural Zambia, in which most adolescent girls reported that they reduced school attendance, participation, and physical movement for fear of revealing blood stains and odor, which would lead to embarrassment for girls and teasing from other students, especially boys [5]. This quantitative analysis reinforces connections in the literature between boys’ teasing and girls’ inability to manage their periods hygienically and safely. A lack of MHM opportunities within schools puts girls at risk of boys’ period teasing, and lead to girls’ negative emotions surrounding menstruation. Policies need to simultaneously tackle boys’ attitudes toward menstruation and improve girls’ access to suitable menstrual hygiene.

In spite of high prevalence of teasing behavior and understanding of periods as embarrassing, boys scored fairly well (on average 60% correct) on a 10-question test on basic biological facts of menstruation. Boys have received information from formal sources like school curricula (63%) and health workers (59%), and occasionally from informal sources like the Internet (26%). The low prevalence of the Internet as a source of information may be due to overall low levels of Internet use among students. This finding contrasts with findings from studies of boys in India [15] and older men from Australia [14], which reported that much of males’ knowledge acquisition is highly informal–through peers, the Internet, or popular media–often resulting in misconceptions, contradictions, and missing information. In sum, the majority of boys in the four schools have received information about periods through formal channels. Encouragingly, the vast majority of boys (84%) were willing and even eager to learn more about periods.

However, we also find evidence of stigma and social norms surrounding periods that may hinder boys’ positive role in MHM reform. Though very few boys associate menarche with marriagebility, a substantial minority (32%) associate menarche with sexual maturity. Regarding attitudes, there are striking differences in boys’ perceptions of appropriate adults with whom girls might discuss periods. Boys overwhelmingly supported girls speaking to female classmates, female teachers, and mothers, but heavily disapproved of girls speaking to male counterparts. This suggests a potential roadblock to engaging boys in female empowerment; boys may want to learn more, but they find it inappropriate to discuss periods with girls. The majority of boys reported personal attitudes and home environments that depict periods as something to be kept hidden. Two-thirds of boys reported that menstruating females in their homes are restricted from daily activities, most often cooking. These results corroborate qualitative evidence in Tanzania on common household proscriptions for menstruating women, usually out of the belief that menstruation is “dirty” or “unclean” [7].

We tested determinants of period teasing, and found significant associations with behaviors surrounding menstruation among boys’ family and friends. First, there is a robust and positive correlation between home restrictions on menstruation and likelihood of reporting period teasing. That is, period stigma at home may translate to negative behaviors at school, suggesting that successful interventions to curb teasing should extend into broader norms in the family and community. Second, boys who report having male friends who engage in period teasing are more likely to report that they themselves have teased. A portion of this correlation is likely attributable to boys’ differential propensity to confess any type of teasing. However, it is conceptually plausible that boys are more likely to engage in teasing if it is normalized or encouraged by their close male friends. This corroborates our results that one in five boys who report that they have teased list peer pressure as a motivation. Taken together, these two results indicate the presence of a social component of period teasing that cannot solely be eliminated by classroom biological education, which should be further explored in research and MHM policy.

Given that the majority of the boys received information from school curricula and health workers, we expect that each school’s reproductive health curriculum significantly influenced boys’ knowledge. Our finding that boys received reproductive and menstrual health education in schools is likely context-specific. At the national educational level, Tanzanian government has been quite proactive in addressing sexual and reproductive health needs for young people, creating policies like the National Multisectoral and Strategic Framework (NMSF) on HIV and AIDS and the national Adolescent Sexual and Reproductive Health Strategy (ASRH) [39]. However there is likely wide variation at the school- and community-level. There is evidence that the new national protocols have not been implemented uniformly across Tanzania, and that implementation depends on school location, resource ability, and individual teachers’ and administrators’ willingness to engage with the topic of menstruation. One study found that among nine co-ed secondary schools in the Kinondoni and Bagamoyo districts of Tanzania, only three provided any form of menstrual health education to boys, though all schools offered such education to girls [18]. As such, we refrain from applying our results on boys’ sources of knowledge to Tanzania as a whole.

The analysis highlights two contrasting forces. On the one hand, the adolescent boys express willingness to learn more about menstruation (84%), judge peer teasing behavior as “immature” (35%), and report that they would intervene (54%) or talk with the perpetrator (23%) if they witnessed period teasing. This is in contrast to the 3.1% of boys who say they would respond by also teasing the girl. It is clear that the majority of adolescent boys do not condone period teasing and are willing to act as positive agents for change.

On the other hand, teasing is a regular occurrence and most of the boys hold negative personal attitudes toward menstruation, which are strongly connected to negative social norms. These negative social factors include perceived impropriety of girls discussing periods with males, peer pressure to tease, and restrictions in the home.

Our results, though limited to correlation, call into question the effectiveness of interventions that only rely on biomedical education or providing MHM products to reduce negative attitudes and teasing. Although the boys in our sample had relatively high biomedical knowledge of menstruation, they maintained negative attitudes, and knowledgeable boys were equally likely to engage in period teasing as their less knowledgeable peers. Even if girls are given pads, boys may simply switch from teasing about stains/odors to teasing about finding pads in girls’ book bags [1, 17, 40] if the root cause (social norms) are not specifically addressed. As a response, we recommend that menstrual health education in schools should expand beyond the purely biomedical and include social programming that aims to change social norms and period stigma.

### Study weaknesses

The current study cannot be interpreted as a representative sample of Tanzania, as the four schools were selected for convenience. Unfortunately, information on the sexual and menstrual health curricula in the sampled schools is unavailable, so we cannot test this proposed connection between school curricula and boys’ menstrual knowledge. Further, it is important to note that our sample was restricted to boys enrolled in secondary school. Our results on the prevalent role of school curricula and health workers in menstruation education is likely different for out-of-school youth. This is especially important given the high rate of out-of-school males of secondary school age in Sub-Saharan Africa (45%, compared to 48.4% for girls in the same ages) in 2018 [41]. No gender-disaggregated statistics were found for Tanzania. In 2016, Tanzania’s out of school rate for boys and girls in secondary school ages was 76% in 2016 [41].

In addition, the written survey method allows for the possibility that respondents feared their answers would be revealed to others in the room, though students were spaced sufficiently apart. This may have contributed to high levels of non-response, particularly on questions about friends and socially sanctioned behaviors. Presence of head teachers in some classrooms may have also caused some degree of social desirability bias in answers, although surveys were filled in in private.

Finally, there are very few previous studies from other countries or regions with which we can compare our results for boys’ overall knowledge, rates of teasing (among boys and girls), and sources of information. As such, it is difficult to conclude whether boys in these schools, or boys in Tanzania, are more, less, or equally informed compared to their counterparts in different national contexts. The only comparable result we found was the MENISCUS-2 trial [19], conducted among 232 girls and 218 boys in Uganda, which found that 30% of girls and 19% of boys scored eight out of nine possible points on a knowledge test, compared to roughly 7% of girls and 10% of boys in our sample. Notably, while girls in the MENISCUS-2 trial consistently score higher than boys, we find no significant gender differences in our sample. However, differences in the structure and content of the two knowledge tests limits numerical comparison of these results (see S1 Appendix for full list of knowledge questions).

### Further research

We recommend four avenues for future research. First, research should investigate how menstruation is taught in schools and seek to quantify the differential effect of various curricula or teaching styles (ex. gender-segregated or co-educational lessons). In particular, such research should distinguish between biomedical teaching of periods and socially-focused programming aiming to change norms and stigma. Second, future work should seek to quantify boys’ knowledge, experience, and attitudes from around the world, employing a comparative approach to the social, environmental, and educational factors affecting menstruation. Third, qualitative research should investigate the intra-household mechanism by which menstrual restrictions are enforced, despite common sentiment that menstruation should not be revealed. Fourth, our results highlight the potential efficacy and necessity of gender-inclusive programs that combine interventions into boys’ attitudes and girls’ material needs.

## Conclusions

Period-related teasing is a major source of anxiety for adolescent girls. Through quantitative surveying, we estimate that 13% of girls enrolled in the four secondary schools have experienced period teasing and that more than 80% fear experiencing it. Boys corroborate these findings as they report engaging in or seeing their friends engage in such behaviors. While the theme of period teasing has surfaced in qualitative research, little emphasis has been placed on the matter in the context of schooling policy and how to reduce gender gaps in education. Ensuring harassment-free schooling environments and access to sanitation remain as challenges to tackle to achieve gender equality in education. To reduce harassment, MHM policymakers should explore complementary interventions to reduce stigma and correct harmful behaviors surrounding menstruation among adolescent boys. The study results indicate that adolescent boys may be willing to engage in menstrual health programming as they express interest in learning more.

## Supporting information

S1 Appendix (PDF)

S1 Dataset (DTA)

S2 Dataset (DTA)

S1 Questionnaires (PDF)
