# Supplementary material for: Period teasing, stigma and knowledge: A survey of adolescent boys and girls in Northern Tanzania
Source: PLoS One. 2020 Oct 28;15(10):e0239914. doi: 10.1371/journal.pone.0239914 (PMC7592731; doi:10.1371/journal.pone.0239914)
Supplement: S1 Appendix — (PDF) [file pone.0239914.s001.pdf]

S1 Appendix

Table 1. Relationship between boys' reported period teasing and specific restrictions on menstruating women in the home

	Coefficient on "Have teased girls about periods" *bin(0/1)* (1)	Sample average (2)
"In my house, menstruating women are restricted from..."		
Touching water or animals	0.123**	0.161
	(2.19)	
Cooking	0.0473	0.293
	(1.03)	
Washing dishes	0.0501	0.156
	(0.86)	
Public gatherings	0.131**	0.179
	(2.48)	
Sleeping in normal spot	0.344***	0.060
	(3.86)	
Using normal latrine	-0.0355	0.087
	(-0.49)	
Other	0.0724	0.069
	(0.91)	
Observations	357	

Notes: Controls (not reported) are age, grade, and menstrual knowledge score. School fixed effects included.

Fig 1. Knowledge Questions

Question	Correct Answer	Correct	Incorrect/DK	NA
Q2: How often does a girl generally get her period?	Once a month	310	120	2
		71.76	27.78	0.46
Q3: How long does avg. period last?	A few days	211	217	4
		49.3	50.23	0.93
Q4: At what age girls generally get first period?	11-16	340	63	29
		78.7	14.58	6.71
Q5: At what age women stop getting periods?	45-55	220	165	47
		50.93	38.19	10.88
Q6a: When girl reaches menarche, she can get pregnant from sex (Y/N/DK)	Y	369	57	6
		85.42	13.19	1.39
Q6b: Menstrual blood is shedding of endometrium (Y/N/DK)	Y	258	161	13
		59.72	37.27	3.01
Q6c: Menstrual period means woman is not pregnant (Y/N/DK)	Y	267	154	11
		61.81	35.65	2.55
Q6d: Ovulation happens 14 days after period (Y/N/DK)	Y	228	180	24
		52.78	41.67	5.56
Q6e: Ovulation happens during period (Y/N/DK)	N	227	142	33
		52.55	32.87	7.63
Q6f: Common for girls to have physical discomfort on period? (Y/N/DK)	Y	274	137	21
		63.43	38.66	4.86
