# Supplementary material for: Period teasing, stigma and knowledge: A survey of adolescent boys and girls in Northern Tanzania
Source: PLoS One. 2020 Oct 28;15(10):e0239914. doi: 10.1371/journal.pone.0239914 (PMC7592731; doi:10.1371/journal.pone.0239914)
Supplement: S1 Questionnaires — (PDF) [file pone.0239914.s004.pdf]

SUPPLEMENTARY MATIERAL
QUESTIONNAIRE for “Period teasing, stigma and knowledge: a survey of adolescent boys and girls in Northern Tanzania” by Benshaul-Tolonen et al. 2020

Surveying date:
School ID:
Classroom ID:
Student ID:

BOYS QUESTIONNAIRE

Dodoso

Kipengele 1: Marafiki

1. Sasa tungependa utuambie kuhusu marafiki 5 wa KIUME wa karibu zaidi ulio nao shuleni. Tafadhali taja majina ya marafiki zako wa karibu kabisa. Taja tu marafiki ambao wanasoma shule hii, na ambao wapo darasa moja na wewe, na unaowahesabu kama marafiki.

Andika majina yao kamili na darasa la marafiki uliowataja

a. ______________________________
b. ______________________________
c. ______________________________
d. ______________________________
e. ______________________________

2. Kwa maoni yako,ni wavulana gani maarufu zaidi shuleni?

Andika majina kamili na darasa la wavulana uliowataja

a. ______________________________
b. ______________________________
c. ______________________________
d. ______________________________
e. ______________________________

Kipengele 2: Taarifa
Tungependa kukuuliza maswali kuhusu hedhi.

1. Kwanini msichana au mwanamke anapata hedhi? Chagua yote yanayokubalika
   a. ☐ dhambi binafsi
   b. ☐ kutokana na nguvu za giza
   c. ☐ kwa sababu hakupata mimba mwezi huo
   d. ☐ kutoa damu mbaya
   e. ☐ mengine
   f. ☐ Sijui

2. Ni mara ngapi msichana (ambaye ameshavunja ungo) anapata hedhi kwa wastani? (Chagua moja)
   ☐ mara moja katika maisha yake
   ☐ mara moja kila mwaka
   ☐ kila wiki
   ☐ mara moja kila mwezi
   ☐ sijui

3. Kwa wastani hedhi huwa inachukua muda gani? (Chagua moja)
   ☐ saa moja

Surveying date:
School ID:
Classroom ID:
Student ID:

---

 siku moja
 siku kadhaa
 wiki mbili
 muda wote

4. Kiujumla ni katika umri gani wasichana huwa wanapata hedhi yao kwa mara ya kwanza?

 ____

5. Katika umri gani wanawake hufikia ukomo wa kupata hedhi?

 ____

6. Chagua jibu sahihi.
 Tafadhali chagua Ndio au Hapana kuashiria kama kauli zifuatazo ni za ukweli.

 a) Wakati msichana akivunja ungo (akipata hedhi kwa mara ya kwanza), iamaanisha anaweza kupata mimba kama atafanya mapenzi.
  Ndio Hapana Sijui
 b) Damu ya kipindi cha hedhi hutokana na kumwaga damu ya ukuta ndani ya tumbo la uzazi
  Ndio Hapana Sijui
 c) Hedhi huashiria kwamba mwanamke hana mimba
  Ndio Hapana Sijui
 d) Kupevuka mayai hutokea kwa wastani siku 14 kabla ya hedhi
  Ndio Hapana Sijui
 e) Kupevuka mayai hutokea kipindi cha hedhi
  Ndio Hapana Sijui
 f) Je, huwa ni kawaida kwa wasichana kujisikia maumivu ya kimwili wakipata hedhi?
  Ndio Hapana Sijui
 g) Je, wasichana hupitia yafuatayo wakiwa hedhini?

Hisia mbaya zisizozuilika [hasira]	Ndio	Hapana	Sijui
Mfadhaiko	Ndio	Hapana	Sijui
Uchovu mkali	Ndio	Hapana	Sijui

Kipengele 3: Uzoefu Binafsi

1. Kuna mtu yeyote amewahi kukuambia kuhusu hedhi na kwanini inatokea?
  NDIO HAPANA

 Kama ndio, nani? Tafadhali chagua zote zinazokubalika.

mama	baba
bibi	babu
dada	kaka

Surveying date:
School ID:
Classroom ID:
Student ID:

☐ rafiki wa kike	☐ rafiki wa kiume
☐ shangazi au ndugu mwingine wa kike	☐ mjomba au ndugu wa kiume
☐ nesi	☐ mchungaji / kiongozi wa kidini
☐ mwalimu wa kike	☐ mwalimu wa kiume
☐ mpenzi/mwenza	☐ mwingine

2. Uliwahi kupokea taarifa kuhusu hedhi kutoka kwa wafuatao:
   a) Mtandaoni ☐ NDIO ☐ HAPANA
   b) Mtoa huduma ya afaya ☐ NDIO ☐ HAPANA
   c) Mtaala wa shule ☐ NDIO ☐ HAPANA
   d) Vipeperushi (matangazo, mabango) ☐ NDIO ☐ HAPANA

3. Ulifanya nini uliposikia kuhusu hedhi mara ya kwanza? (Tafadhali chagua moja kutoka kila kundi la hizo 3, ambapo 2 ni katikati.)

	**1**	**2**	**3**	
ulishangaa	☐	☐	☐	hukushangaa
uliogopa	☐	☐	☐	hukuogopa
ulipata msisimko	☐	☐	☐	hukusisimka
ulichanganyikiwa	☐	☐	☐	hukuchanganyikiwa
ulijisikia ujasiri	☐	☐	☐	hukujisikia jasiri
ulisikia kinyaa	☐	☐	☐	hukusikia kinyaa
ulijivunia	☐	☐	☐	hukujivunia
ulijionea haya	☐	☐	☐	hukujionea haya

Kipengele 4: Uratibu wa Hedhi kwa wanarika

1. Tafadhali kadiria kiasi cha wanafunzi wenzako wa kike ambao tayari wamevunja ungo Utapokea dola 1 ya kimarekani ukikisia jibu sahihi.

Surveying date:
School ID:
Classroom ID:
Student ID:

☐ 0%
☐ 1-25%
☐ 26-50%
☐ 51-75%
☐ 76-100%

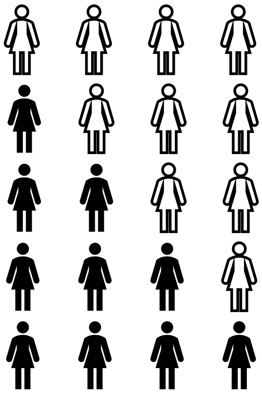

2. Mara nyingine wasichana hutaniwa kuhusu hedhi. Tafadhali kadiria kiasi cha wanafunzi wenzako wa kike ambao wamewahi kutaniwa kuhusu hedhi. Utapokea dola 1 ya kimarekani ukikisia jibu sahihi.

☐ 0%
☐ 1-25%
☐ 26-50%
☐ 51-75%
☐ 76-100%

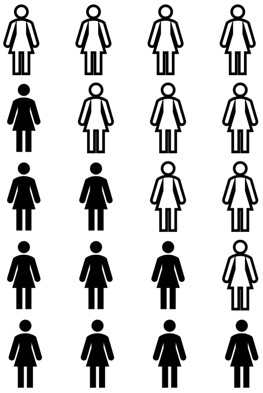

3. Tafadhali kisia njia **inayotumika zaidi** ya kujiweka safi miongoni mwa watu wa rika lako darasani. Tafadhali chagua moja.

☐ kitambaa	☐ pedi
☐ tampuni	☐ bidhaa asilia (e.g. nyasi, majani, mavi ya ng'ombe)
☐ vigodoro	☐ kikombe cha hedhi
☐ hawatumii chochote	☐ nyingine ___________________________________

3. Unajiamini kiasi gani kwamba unajua nini wanafunzi wenzio wanatumia wakiwa hedhini?
   Sijiamini kabisa 1 2 3 4 5 Najiamini kabisa

4. Kama wanatumia pedi, unajua namna gani wanavyozitupa/[wanavyozitokomeza zikishatumika]?
   ☐ NDIO ☐ HAPANA

5. Kama ulijibu ndio kwenye swali lililopita, ni njia ganiwanayoitumia zaidi?
   ☐ hawatupi (wanatumia zaidi ya mara moja)
   ☐ ndoo ya taka
   ☐ choo
   ☐ kuzichoma moto

Surveying date:
School ID:
Classroom ID:
Student ID:

---

 kuzizika
 nyingine ____

Kipengele 5: Mienendo juu ya Hedhi

1. Unajisikia salama kwenye vyoo vya shule?
 NDIO HAPANA

2. Huwa unajua wakati wanafunzi wenzako wa kike wakiwa hedhini?
 NDIO HAPANA

3. Unadhani wasichana wangejisikia aibu endapo utajua kwamba wapo hedhini?
 NDIO HAPANA

4. Je, unahisi kwamba kupata hedhi ni kitu ambacho kinapaswa kuwekwa siri shuleni?
 NDIO HAPANA

5. Unafikiri wasichana wanaogopa kuwaambia watu kuhusu hedhi zao?
 NDIO HAPANA

6. Je, ni sahihi kwa wasichana kuzungumza kuhusu hedhi zao mbele za watu?
 Ni sahihi kabisa Sahihi Katikati Sio sahihi Sio sahihi kabisa

7. Nini kingetokea kama msichana angeongelea hedhi yake mbele za watu?
____

8. Mara nyingine huwa inajalisha nani msichana ataongea naye kuhusu hedhi yake.

a. Je, ni sahihi kwa wasichana kuongea kuhusu hedhi zao na wanafunzi wenzao wa kike?
 Ni sahihi kabisa Sahihi Katikati Sio sahihi Sio sahihi kabisa

b. Je, ni sahihi kwa wasichana kuongea kuhusu hedhi zao na wanafunzi wenzao wa kiume?
 Ni sahihi kabisa Sahihi Katikati Sio sahihi Sio sahihi kabisa

c. Je, ni sahihi kwa wasichana kuongea kuhusu hedhi zao na mwalimu wa kike?
 Ni sahihi kabisa Sahihi Katikati Sio sahihi Sio sahihi kabisa

d. Je, ni sahihi kwa wasichana kuongea kuhusu hedhi zao na mwalimu wa kiume?
 Ni sahihi kabisa Sahihi Katikati Sio sahihi Sio sahihi kabisa

e. Je, ni sahihi kwa wasichana kuongea kuhusu hedhi zao na baba zao?
 Ni sahihi kabisa Sahihi Katikati Sio sahihi Sio sahihi kabisa

f. Je, ni sahihi kwa wasichana kuongea kuhusu hedhi zao na mama zao?
 Ni sahihi kabisa Sahihi Katikati Sio sahihi Sio sahihi kabisa

Surveying date:
School ID:
Classroom ID:
Student ID:

---

9. Je, unakubaliana na kauli ifuatayo: "Nyumbani kwetu wanawake/wasichana lazima waone aibu kuongelea au kuweka wazi hedhi yao (damu, maumivu, vifaa vya kujiweka safi) mbele ya wanafamilia."
   ☐ nakubaliana sana ☐ nakubaliana ☐ katikati ☐ sikubaliani ☐ sikubaliani kabisa

10. Unakubaliana na kauli ifuatayo: "Nyumbani kwetu, wanawake na wasichana ambao wako hedhini wanazuiliwa kufanya shughulli Fulani."
    ☐ NDIO ☐ HAPANA ☐ Sijui

   Kama ulijibu NDIO, shughuli gani? Chagua zote zinazokubalika

   a) ☐ Kushika vyanzo vya maji au wanyama
   b) ☐ Kupika
   c) ☐ Kuosha vyombo
   d) ☐ Mikutano ya hadhara kama vile mikutano ya kidini, mikutano ya jumuiya
   e) ☐ Kulala sehemu yake ya kila siku
   f) ☐ Kutuia choo ambacho uwa anatumia siku zote
   g) ☐ Nyingine: ______________________________

11. Ungependa kuwa namtu ambaye ungeweza kumuuliza kuhusu hedhi?
    ☐ NDIO ☐ HAPANA

Kipengele 6: Teasing

1. Kutoka kwenye wale marafiki wa KIUME uliowaandika juu (Kipengele 1, Swali 1), **wangapi** umewaona wakiwatania wasichana kuhusu hedhi zao?
   ☐ Hakuna ☐ Rafiki 1 ☐ Marafiki 2 ☐ Marafiki 3 ☐ Marafiki 4 ☐ Marafiki 5

2. Kutoka wale marafiki uliowataja juu, ni wangapi kati yao umeshawahi kujadili masuala ya hedhi?
   ☐ Hakuna ☐ Rafiki 1 ☐ Marafiki 2 ☐ Marafiki 3 ☐ Marafiki 4 ☐ Marafiki 5

3. Je, marafiki wako wa karibu huwa wanawatania wasichana kuhusu hedhi? (kama Kamwe/Sijui, ruka hadi Swali 6)
   ☐ Daima ☐ Mara nyingi ☐ Mara chache ☐ Kamwe ☐ Sijui

4. Kama ndio, huwa inatokea muda gani?
   ☐ Wakiwa wanatoa harufu mbaya
   ☐ Pindi nguo zao zikiwa na madoa ya damu
   ☐ Pale wakiona pedi au vifaa vingine miongoni mwa mali za wasichana
   ☐ Wakati wasichana wakiwa hawataki kushiriki kwenye michezo kwa sababu ya hedhi
   ☐ Wakihisi kwamba msichana yuko hedhini

   ☐ Mengineyo: ______________________________

Surveying date:
School ID:
Classroom ID:
Student ID:

---

5. Unafikiri kwa nini marafiki zako wanawatania wasichana kuhusu hedhi zao?
 Kwa sababu hedhi inaaibisha
 Kwa sababu hedhi ni kitu kisicho cha asili
 Kwa sababu hedhi inatokana na nguvu za giza
 Kwa sababu hedhi ni dhambi
 Kwa sababu wavulana wana mambo ya kitoto, au hawazielewi hedhi

 Mengineyo: ____

6. Umewahi kumtania msichana kuhusu hedhi?
 NDIO HAPANA

7. Kama NDIO, lini? Chagua yote yanayokubalika.
 Wakiwa wananuka vibaya
 Pindi nguo zao zikipata madoa ya damu
 Pale nikiona pedi au vifaa vingine miongoni mwa mali za wasichana
 Wakati wasichana wakiwa hawataki kushiriki kwenye michezo kwa sababu ya hedhi
 Nikihisi kwamba msichana yuko hedhini

 Mengineyo: ________________________________

8. Kama NDIO kwanini uliwatania?
 Kwa sababu hedhi inaaibisha
 Kwa sababu hedhi ni kitu kisicho cha asili
 Kwa sababu hedhi inatokana na nguvu za giza
 Kwa sababu hedhi ni dhambi
 Kwa sababu wavulana wengine pia walikuwa wanatania

 Mengineyo: ____

9. Lini mara ya mwisho umemtania msichana kuhusu hedhi?
 Ndani ya wiki iliyopita
 Ndani ya mwezi uliopita
 Ndani ya miezi 6 iliyopita
 Ndani ya mwaka uliopita
 Zaidi ya mwaka mmoja uliopita

10. Lini mara ya mwisho umeona **mwanafunzi mwenzako** anamtania msichana kuhusu hedhi?
 Ndani ya wiki iliyopita
 Ndani ya mwezi uliopita
 Ndani ya miezi 6 iliyopita
 Ndani ya mwaka uliopita
 Zaidi ya mwaka mmoja uliopita

11. Unafanya nini ukina mwanafunzi mwenzako anamtania msichana kuhus hedhi yake?

Surveying date:
School ID:
Classroom ID:
Student ID:

☐ Sifanyi chochote
☐ Nazungumza na mwanafunzi mwenzangu huyo aliyetania tukiwa faragha
☐ Nazungumza na msichana aliyetaniwa tukiwa faragha
☐ Namwambia mwanafunzi mwezangu aache
☐ Mimi pia namtania huyo msichana
☐ Namwambia mwalimu au mtu mzima mwingine

☐ Mengineyo ______________________

Kipengele 7: Wenza

1. Umewahi kuwa na mpenzi?
   ☐ NDIO ☐ HAPANA

2. Je, una mpenzi hivi sasa?
   ☐ NDIO ☐ HAPANA (nenda swali 31)

3. Mpenzi wako ana umri gani?
   ____

4. Je ni sawa kwa msichana kumuomba mpenzi wake kulipia bidahaa?
   ☐ NDIO ☐ HAPANA

   30a. Kama ndio, nini? (chagua zote zinazokubalika)
   ☐ nguo ☐ chakula ☐ pedi ☐ mengineyo ______

5. Je ni sawa kwa mvulana kutarajia wasichana watafanya naye mapenzi baada ya kuwapa zawadi au kulipia bidhaa?
   ☐ NDIO ☐ HAPANA

6. Unakubali kwamba wasichana lazima waolewe mara tu baada ya kupata hedhi.
   ☐ nakubaliana sana ☐ nakubaliana ☐ katikati ☐ sikubaliani ☐ sikubaliani kabisa

7. Unakubali kwamba wasichana wanakuwa wakubwa kiasi cha kutosha kufanya mapenzi baada ya kupata hedhi
   ☐ nakubaliana sana ☐ nakubaliana ☐ katikati ☐ sikubaliani ☐ sikubaliani kabisa

Kipengele 8: Maswali juu ya Vitu Tunavyovijali

1. Wazazi/walezi wangu wananihamasisha kufanya vyema shuleni
   ☐ nakubaliana sana ☐ nakubaliana ☐ katikati ☐ sikubaliani ☐ sikubaliani kabisa

2. Walimu wangu hunitia moyo na kunipa msaada
   ☐ nakubaliana sana ☐ nakubaliana ☐ katikati ☐ sikubaliani ☐ sikubaliani kabisa

Surveying date:
School ID:
Classroom ID:
Student ID:

---

3. Elimu ni ya muhimu zaidi kwa wavulana kuliko kwa wasichana
   ▯ nakubaliana sana ▯ nakubaliana ▯ katikati ▯ sikubaliani ▯ sikubaliani kabisa

4. Huko mbeleni mambo yatakuwa sawa kwangu
   ▯ nakubaliana sana ▯ nakubaliana ▯ katikati ▯ sikubaliani ▯ sikubaliani kabisa

5. Nina wasiwasi karibu muda wote
   ▯ nakubaliana sana ▯ nakubaliana ▯ katikati ▯ sikubaliani ▯ sikubaliani kabisa

6. Nimeridhika na maisha yangu
   ▯ nakubaliana sana ▯ nakubaliana ▯ katikati ▯ sikubaliani ▯ sikubaliani kabisa

Kipengele 9: Demgrafia

1. Jina lako nani? (la kwanza na la pili)
   ____

2. Umeishi muda gani katika makazi uliyopo? ______________________

3. Umezaliwa mwezi gani na mwaka gani?____

4. Ulifikisha miaka mingapi kwenye tarehe yako ya kuzaliwa iliyopita?
   ____

5. Upo darasa la ngapi?____

6. Ulianza shul ukiwa na umri gani? ____

7. Tafadhali chagua kabila lako linalokutambulisha
   ▯ Mchagga
   ▯ Mmasai
   ▯ Msukuma
   ▯ Mhaya
   ▯ Msumbwa
   ▯ kabila linginelo:_____________________________________

8. Tafadhali chagua dhehebu linalokutambulisha
   ▯ Mkristo
   ▯ Muislamu
   ▯ Dhehebu jinginelo:______________________________________
   ▯ Sina dhehebu

9. Unaishi na nani kwa sasa?
   ▯ wazazi ▯ bibi na babu ▯ peke yangu
   ▯ ndugu niliozaliwa nao ▯ wanafamilia wengine ▯ bweni la shule
   ▯ bweni/hosteli binafsi ▯ nyingine __________________________

Surveying date:
School ID:
Classroom ID:
Student ID:

---

10. Unaweza kuoga nyumbani?
    ☐ NDIO ☐ HAPANA

11. Unatumia jumla ya kiasi gani cha pesa kwa mwezi? ___________

12. Chanzo chako kikuu cha pesa ni kipi? Tafadhali chagua moja.
    ☐ mama ☐ baba ☐ mpenzi
    ☐ dada / kaka ☐ nyingine ☐ kazi
    ☐ ufadhili

13. Huwa unaitumia [hela] kufanya nini? Tafadhali chagua matumizi makuu matatu.
    ☐ nguo
    ☐ usafi wa mwili
    ☐ vipodozi
    ☐ simu ya mkononi
    ☐ nywele
    ☐ vifaa vya shule
    ☐ chakula
    ☐ usafiri
    ☐ nyingine

14. Je, umekosa kwenda shule ndani ya siku 7 zilizopita?
    ☐ NDIO ☐ HAPANA

Kama Ndio, sababu ilikuwa nini? ____

Surveying date:
School ID:
Classroom ID:
Student ID:

GIRLS QUESTIONNAIRE

Dodoso

Kipengele 1: Marafiki

1. Tungependa utuambie kuhusu marafiki zako 5 wa KIKE wa karibu kabisa ulionao shuleni. Tafadhali taja majina ya marafiki zako wa karibu kabisa. Taja tu marafiki ambao wanasoma shule hii, na ambao wako darasa moja na wewe, na ambao unawahesabu kama marafiki wa karibu.

Andika majina kamili na darasa la marafiki uliowataja

a. ____________________________________
b. ____________________________________
c. ____________________________________
d. ____________________________________
e. ____________________________________

2. Sasa tungependa utuambie kuhusu marafiki wa KIUME wa karibu sana ulio nao shuleni. Tafadhali yataje majina ya marafiki zako wa karibu kabisa. Taja tu marafiki ambao wanasoma shule hii, na ambao wako darasa moja na wewe, na ambao unawahesabu kama marafiki wa karibu.

Andika majina kamili na darasa la marafiki uliowataja

a. ____________________________________
b. ____________________________________
c. ____________________________________
d. ____________________________________
e. ____________________________________

Kipengele 2: Taarifa
Tungependa kukuuliza maswali kadhaa kuhusu hedhi.

1. Kwanini msichana au mwanamke anapata hedhi? Chagua yote yanayokubalika.
   - ☐ dhambi binafsi
   - ☐ kutokana na nguvu za giza
   - ☐ kwa sababu hakupata mimba hedhi huo
   - ☐ kutoa damu mbaya
   - ☐ mengineyo
   - ☐ sijui

2. Ni mara ngapi msichana (ambaye tayari ameshapata hedhi) anapata hedhi kwa wastani?
   - ☐ mara moja katika maisha yake
   - ☐ mara moja kila mwaka
   - ☐ kila wiki
   - ☐ kila hedhi
   - ☐ sijui

Surveying date:
School ID:
Classroom ID:
Student ID:

---

3. Kwa wastani hedhi huwa inachukua muda gani? Chagua moja.
   - ☐ saa moja
   - ☐ siku moja
   - ☐ siku kadhaa
   - ☐ wiki mbili
   - ☐ muda wote
   - ☐ sijui

4. Kiujumla ni katika umri gani wasichana huwa wanapata hedhi yao kwa mara ya kwanza?

   ____

5. Katika umri gani wanawake hufikia ukomo na kupata hedhi?

   ____

6. Chagua jibu sahihi
   Tafadhali chagua Ndio au Hapana kuashiria kama kauli zifuatazo ni za ukweli.

   a) Msichana akivunja ungo (kupata hedhi kwa mara ya kwanza), inamaanisha anaweza kupata mimba akifanya mapenzi.
   ☐ Ndio ☐ Hapana ☐ Sijui
   b) Damu ya kipindi cha hedhi hutokana na kumwaga damu ya ukuta ndani ya tumbo la uzazi
   ☐ Ndio ☐ Hapana ☐ Sijui
   c) Hedhi huashiria kwamba mwanamke hana mimba
   ☐ Ndio ☐ Hapana ☐ Sijui
   d) Kupevuka mayai hutokea kwa wastani siku 14 kabla ya hedhi
   ☐ Ndio ☐ Hapana ☐ Sijui
   e) Kupevuka mayai hutokea kipindi cha hedhi
   ☐ Ndio ☐ Hapana ☐ Sijui
   f) Je, huwa ni kawaida kwa wasichana kujisikia maumivu ya kimwili wakipata hedhi?
   ☐ Ndio ☐ Hapana ☐ Sijui
   g) Je, wasichana wengi hupitia yafuatayo wakiwa hedhini?

Hisia mbaya zisizozuilika [hasira]	☐ Ndio	☐ Hapana	☐ Sijui
Mfadhaiko	☐ Ndio	☐ Hapana	☐ Sijui
Uchovu mkali	☐ Ndio	☐ Hapana	☐ Sijui

Kipengele 3: Uzoefu Binafsi

1. Kuna mtu yeyote amewahi kukuambia kuhusu hedhi na kwanini inatokea?
   ☐ NDIO ☐ HAPANA

   Kama ndio, nani? Tafadhali chagua zote zinazokubalika.
   ☐ mama ☐ baba

Surveying date:
School ID:
Classroom ID:
Student ID:

☐ bibi	☐ babu
☐ dada	☐ kaka
☐ Rafiki wa kike	☐ rafiki wa kiume
☐ shangazi au ndugu wa kike	☐ mjomba au ndugu wa kiume
☐ nesi	☐ mchungaji / kiongozi wa kidini
☐ mwalimu wa kike	☐ mwalimu wa kiume
☐ mpenzi/mwenza	☐ mwingine

2. Uliwahi kupokea taarifa kuhusu hedhi kutoka kwa wafuatao:

• Mtandaoni	☐ NDIO	☐ HAPANA
• Mtoa huduma ya afya	☐ NDIO	☐ HAPANA
• Machapisho ya shuleni (vitabu, madarasa, homework, n.k.)	☐ NDIO	☐ HAPANA
• Vipeperushi (matangazo, mabango)	☐ NDIO	☐ HAPANA

3. Ulifanya nini uliposikia kuhusu hedhi mara ya kwanza? (Tafadhali chagua moja kutoka kila kundi la hizo 3, ambapo 2 ni katikati.)

	1	2	3	
ulishangaa	☐	☐	☐	sikushangaa
uliogopa	☐	☐	☐	sikuogopa
ulipata msisimko	☐	☐	☐	hukusisimka
ulichanganyikiwa	☐	☐	☐	hukuchanganyikiwa
ulijisikia ujasiri	☐	☐	☐	hukujisikia jasiri
ulisikia kinyaa	☐	☐	☐	hukusikia kinyaa
ulijivunia	☐	☐	☐	hukujivunia
ulijionea haya	☐	☐	☐	hukujionea haya

4. Kuna mtu amewahi kukuambia nini unapaswa kufanya ukianza kupata hedhi?
☐ NDIO ☐ HAPANA

Kama ndio, ulipokea ushauri gani? Tafadhali chagua yote yanayokubalika.

Surveying date:
School ID:
Classroom ID:
Student ID:

☐ namna ya kutumia taulo/vitambaa/vitambaa vya pamba
☐ namna ya kubakia msafi/kunawa
☐ namna ya kumudu mauimivu
☐ mambo/shughuli za kuepuka wakati wa hedhi
☐ mengineyo ___________

5. Weka alama kwa watu wote ambao UNGEPENDA kuongea nao kuhusu hedhi yako.

☐ mama	☐ baba
☐ bibi	☐ babu
☐ dada	☐ kaka
☐ rafiki wa kike	☐ rafiki wa kiume
☐ shangazi au ndugu mwingine wa kike	☐ mjomba au ndugu wa kiume
☐ nesi	☐ mchungaji / kiongozi wa kidini
☐ mwalimu wa kike	☐ mwalimu wa kiume
☐ mpenzi/mwenza	☐ mwingine
☐ hakuna hata mtu mmoja	

6. Na nani USINGEPENDA KABISA kuongea naye kuhusu hedhi yako. Tafadhali chagua yote yanayokubalika.

☐ mama	☐ baba
☐ bibi	☐ babu
☐ dada	☐ kaka
☐ rafiki wa kike	☐ rafiki wa kiume
☐ shangazi au ndugu mwingine wa kike	☐ mjomba au ndugu wa kiume
☐ nesi	☐ mchungaji / kiongozi wa kidini
☐ mwalimu wa kike	☐ mwalimu wa kiume
☐ mpenzi/mwenza	☐ mwingine
☐ hakuna hata mtu mmoja	

7. Umeshapata hedhi yako?
   ☐ Ndio **(endelea swali linalofuata)** ☐ Hapana **(nenda Kipengele 4, Swali 1)**

8. Katika umri gani ulipata hedhi mara ya kwanza? _______

9. Je, ulipopata hedhi mara ya kwanza ilikuwa kitu cha hali ya chanya, hasi, au hali tu ya kawaida?
   ☐ chanya ☐ hasi ☐ katikati

10. Ulijisikiaje ulipopata hedhi kwa mara ya kwanza? Ulikuwa…
(Tafadhali chagua moja kutoka kila kundi la hizo 3, ambapo 2 ni katikati.)

	1	2	3	
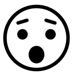 ulishangaa	☐	☐	☐	sikushangaa

Surveying date:
School ID:
Classroom ID:
Student ID:

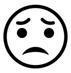	uliogopa	☐	☐	☐	sikuogopa
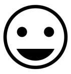	ulipata msisimko	☐	☐	☐	hukusisimka
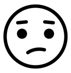	ulichanganyikiwa	☐	☐	☐	hukuchanganyikiwa
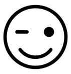	ulijisikia ujasiri	☐	☐	☐	hukujisikia jasiri
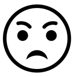	ulisikia kinyaa	☐	☐	☐	hukusikia kinyaa
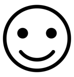	ulijivunia	☐	☐	☐	hukujivunia
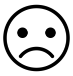	ulijionea haya	☐	☐	☐	hukujionea haya

11. Fikiria nyuma hadi muda kabla hujapata hedhi. Mtu yeyote alikuambia kuhusu hedhi kabla haijaanza?

☐ NDIO ☐ HAPANA

Kama ndio, nani? Tafadhali chagua zote zinazokubali.

☐ mama ☐ baba
☐ bibi ☐ babu
☐ dada ☐ kaka
☐ rafiki wa kike ☐ rafiki wa kiume
☐ shangazi au ndugu mwingine wa kike ☐ mjomba au ndugu wa kiume
☐ nesi ☐ mchungaji / kiongozi wa kidini
☐ mwalimu wa kike ☐ mwalimu wa kiume
☐ mpenzi/mwenza ☐ mwingine

12. Hedhi yako huwa inadumu kwa siku ngapi/Hedhi yako huwa inachukua muda gani kuisha?
siku ____________

13. Kwenye kipimo cha 1 hadi 5 ambapo 1 ni hakuna maumivu na 5 ni maumivu makali sana; Huwa unapata maumivu ya kimwili kiasi gani ukiwa kwenye hedhi?

	1	2	3	4	5	
Sipati maumivu kabisa	☐	☐	☐	☐	☐	*Maumivu makali sana*

14. Kwenye kipimo cha 1 hadi 5 ambapo 1 ni kutokwa damu kidogo na 5 ni kutokwa damu nyingi; Unatoka damu kiasi gani ukiwa hedhini?

	1	2	3	4	5	
Natokwa damu kidogo	☐	☐	☐	☐	☐	*Natokwa damu nyingi*

Surveying date:
School ID:
Classroom ID:
Student ID:

---

15. Kwenye kipimo cha 1 hadi 5, ambapo 1 ni maumivu machache na 5 ni maumivu zaidi na 3 ni sawa; Unalinganisha vipi maumivu ambayo wewe unajisikia ukipata hedhi na ambayo wanayapata wasichana wa rika lako?

	**1**	**2**	**3**	**4**	**5**	
Ninapata maumivu kidogo kuliko wao	☐	☐	☐	☐	☐	*Ninapata maumivu mengi zaidi*

16. Kwenye kipimo cha 1 hadi 5 ambapo 1 ni kutokwa damu kidogo zaidi na 5 ni damu nyingi zaidi na 3 ni sawa; Unafikiri wewe unatokwa damu nyingi zaidi, kidogo zaidi, au sawa na wenzako?

	**1**	**2**	**3**	**4**	**5**	
Ninatokwa damu kidogo zaidi	☐	☐	☐	☐	☐	*Ninatokwa damu nyingi zaidi*

17. Kipindi cha hedhi kinadumu kuanzia siku ya kwanza ya hedhi (kutokwa damu) hadi siku moja kabla ya mwanzo wa kipindi kingine cha hedhi (siku ya kwanza kutokwa damu). Unajua wewe mzunguko wa hedhi yako unachukua muda gani kwa wastani? Kwa maana nyingine, huwa unatunza kumbukumbu ya kwamba unatokwa damu baada ya muda gani?
    ☐ NDIO ☐ HAPANA

18. Unafikiri kwamba kwako muda kati ya hedhi na hedhi unatofautiana sana?
    ☐ NDIO ☐ HAPANA

19. Je, inakuwia vigumu kujua kwamba utapata hedhi hivi karibuni na kujiandaa?
    ☐ NDIO ☐ HAPANA

20. Ukiwa hedhini, au punde tu kabla, huwa unapata…:

Hisia mbaya zisizozuilika [hasira]	☐ NDIO	☐ HAPANA
Mfadhaiko au woga?	☐ NDIO	☐ HAPANA
Uchovu mkali?	☐ NDIO	☐ HAPANA

21. Huwa unatumia dawa za maumivu? (kama vile paracetamol) kumudu maumivu ukipata hedhi?
    ☐ Daima ☐ Mara moja moja ☐ Mara chache ☐ Kamwe

Kipengele 5: Bidhaa Za Hedhi

Surveying date:
School ID:
Classroom ID:
Student ID:

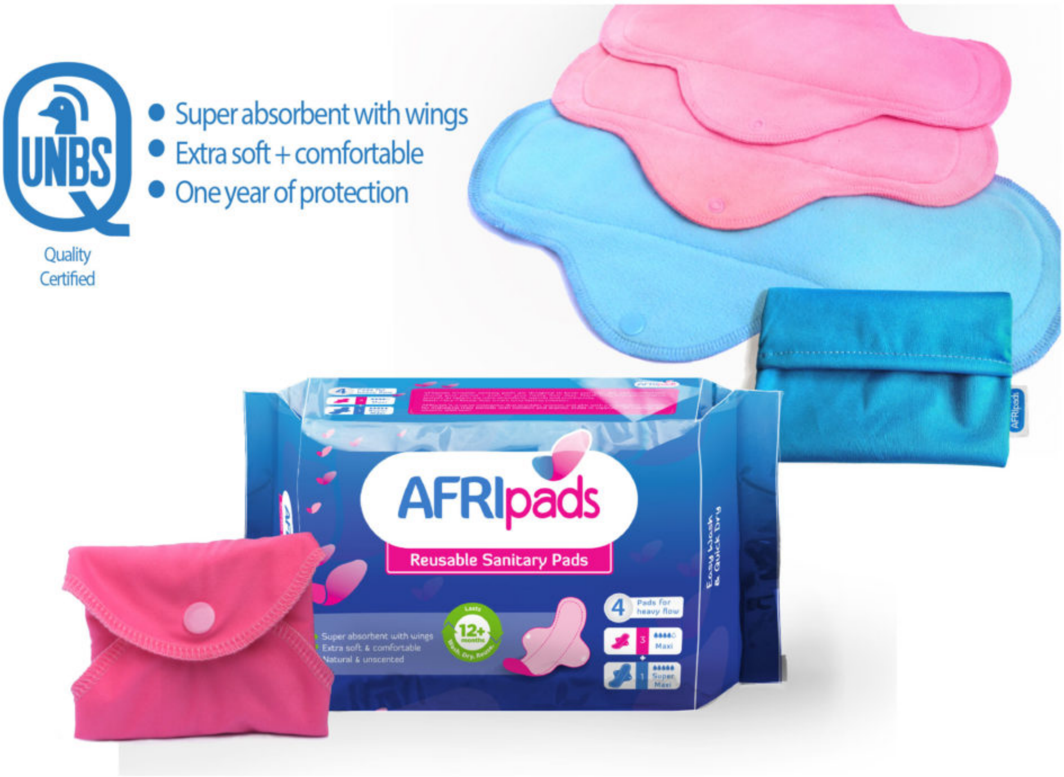

Pedi za kusafisha zinazotumika zaidi ya mara moja ni kitambaa kinachovaliwa kwenye chupi (nguo ya ndani) kuzuia damu ya hedhi kuvujia kwenye nguo. Inaweza kuoshwa, kukaushwa na kutumiwa kwa mwaka 1. Picha hapo juu inaonesha Afripads, aina mojawapo ya pedi za kutumiwa zaidi ya mara moja. Kila pakiti ina pedi 4.

1. Je umewahi Kuona ama Kusikia kuhusu pedi za kusafisha zinazotumika Zaidi ya mara moja?

   ☐ Ndio ☐ Hapana

2. Je umewahi Kutumia pedi za kusafisha zinazotumika Zaidi ya mara moja?

   ☐ Ndio ☐ Hapana

3. Huwa unatumia nini **mara moja moja** ukiwa hedhini? Tafadhali chagua **<u>yote yanayokubalika.</u>**

   ☐ kitambaa ☐ pedi
   ☐ tampuni ☐ bidhaa asilia (e.g. nyasi, majani, mavi ya ng'ombe)
   ☐ vigodoro ☐ kikombe cha hedhi
   ☐ situmii chochote ☐ nyingine __________
   ☐ pedi za kusafisha

4. Kipi huwa unakitumia **mara nyingi** ukiwa hedhini? Tafadhali chagua **<u>moja tu.</u>**

Surveying date:
School ID:
Classroom ID:
Student ID:

☐ kitambaa ☐ pedi
☐ tampuni ☐ bidhaa asilia (e.g. nyasi, majani, mavi ya ng'ombe)
☐ vigodoro ☐ kikombe cha hedhi
☐ situmii chochote ☐ nyingine _________
☐ pedi za kusafisha

5. Je, umewahi kutumia kitambaa?
   ☐ NDIO ☐ HAPANA
   a. Kama umewahi kutumia kitambaa, huwa unafua na sabuni vitambaa unavyotumia ukiwa hedhini?
   ☐ NDIO ☐ HAPANA ☐ Situmii kitambaa
   b. Kama huwa unatumia kitambaa, huwa unavikausha juani vitambaa ulivyovifua?
   ☐ NDIO ☐ HAPANA ☐ Situmii kitambaa
   Kama hapana, wapi ____

6. Ni njia ipi ya kujiweka safi unaipendelea zaidi? Tafadhali <u>chagua moja.</u>
   ☐ kitambaa ☐ pedi
   ☐ tampuni ☐ bidhaa asilia (e.g. nyasi, majani, mavi ya ng'ombe)
   ☐ vigodoro ☐ kikombe cha hedhi
   ☐ situmii chochote ☐ nyingine _________
   ☐ pedi za kusafisha

7. Je, daima huwa unatumia hiyo njia unayoipendelea zaidi?
   ☐ NDIO ☐ HAPANA

8. Fikiria hedhi mmoja ambapo haukutumia hiyo njia unayoipendelea zaidi. Kwanini **HAUKUTUMIA** njia unayoipendelea zaidi?
   ☐ hakuna hela ya kutosha kununua ☐ njia hiyo ilikuwa haipatikani
   ☐ kukosa ruhusa ya wazazi ☐ Sikujua jinsi ya kuitumia [kuipata?]
   ☐ niliona aibu ☐ hiyo njia haikuwa salama
   ☐ nyingine ______________________________

9. Ni mara ngapi unabadilisha kifaa cha kukuweka safi kila siku unapokuwa hedhini?
   ____

10. Unatupaje vifaa vya kujiweka safi **(ile uliyoichagua kwenye swali 24)**?
    ☐ Siitupi (naitumia kwa kuirudia) ☐ ndoo ya taka
    ☐ chooni ☐ naichoma
    ☐ naizika ☐ nyingine ___________________________

Kipengele 5: Uratibu wa Hedhi kwa wanarika

1. Tafadhali kadiria kiasi cha wanafunzi wenzako wa kike ambao tayari wamevunja ungo.

   ☐ 0% 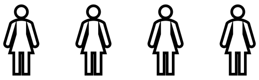

Surveying date:
School ID:
Classroom ID:
Student ID:

☐ 1-25%
☐ 26-50%
☐ 51-75%
☐ 76-100%

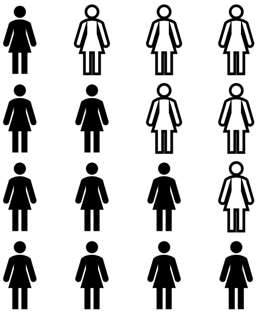

2. Mara nyingine wasichana hutaniwa kuhusu hedhi. Tafadhali kadiria kiasi cha wanafunzi wenzako wa kike ambao wamewahi kutaniwa kuhusu hedhi.

☐ 0%
☐ 1-25%
☐ 26-50%
☐ 51-75%
☐ 76-100%

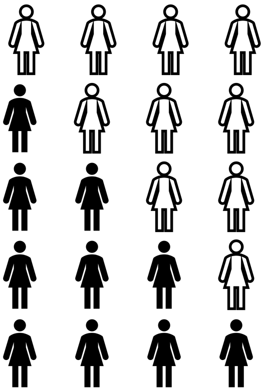

3. Tafadhali kadiria njia **inayotumika zaidi** ya kujiweka safi miongoni mwa watu wa rika lako darasani. Tafadhali chagua moja.

☐ kitambaa
☐ tampuni
☐ vigodoro
☐ situmii chochote
☐ pedi za kusafisha
☐ pedi
☐ bidhaa asilia (e.g. nyasi, majani, mavi ya ng'ombe)
☐ kikombe cha hedhi
☐ nyingine _________

4. Unajiamini kiasi gani kwamba unajua nini wanafunzi wenzio wanatumia wakiwa hedhini?

Sijiamini kabisa ☐1 ☐2 ☐3 ☐4 ☐5 *Najiamini kabisa*

5. Kama wanatumia pedi, unajua namna wanavyozitupa/[wanavyozitokomeza zikishatumika]?

☐ NDIO ☐ HAPANA

6. Kam ulijibu ndio kwenye swali lililopita, ni njia gani wanayoitumia zaidi?

☐ hawatupi (wanatumia zaidi ya mara moja)
☐ ndoo ya taka
☐ choo
☐ kuzichoma moto
☐ kuzizika
☐ nyingine ____

Surveying date:
School ID:
Classroom ID:
Student ID:

Kipengele 6: Utafiti juu ya Shule

1. Unajisikia salama kwenye vyoo vya shule?
    NDIO HAPANA

2. Huwa unajisikia salama kwenye vyoo vya shule ukiwa hedhini?
    NDIO HAPANA

3. Huwa unajua wakati wanafunzi wenzako wa kike wakiwa hedhini?
    NDIO HAPANA

4. Je, unahisi kama kupata hedhi ni kitu ambacho unapaswa kukificha?
    NDIO HAPANA

5. Je, ungesikia aibu kama wasichana wengine wangegundua kwamba upo hedhini?
    NDIO HAPANA

6. Je, ungesikia aibu kama wavulana wangegundua kwamba upo hedhini?
    NDIO HAPANA

7. Kama watu wangejua kwamba umewahi kupata hedhi, ungeogopa kutaniwa?
    NDIO HAPANA
   Kama ndio, nani? Tafadhali chagua yote yanayokubali.
    wasichana wa rika lako wavulana wa rika lako walimu
    hakuna yoyote mwingine ______

8. Kama watu wangejua kwamba umewahi kupata hedhi, ungeogopa kuguswa kinyume na matakwa yako, au kusisitizwa kutoka na mtu fulani?
    NDIO HAPANA
   Kama ndio, na nani? Tafadhali chagua yote yanayokubali.
    vijana wenzio walimu wanafamilia hakuna mtu
    wengine ______

   Kama ndio, una wasiwasi hii itatokea wapi?
    vyoo vya kutumiwa na umati
    vyoo vya shule
    darasani
    viwanja vya shule
    nikiwa njiani kwenda shuleni au kurudi
    nafasi za umma (kama vile viwanja vya umma)
    nyumbani
    sio popote
    nyingine __________________________

9. Kama watu wangejua kwamba umewahi kupata hedhi, ungeogopa mimba isiyopangwa au kutia aibu familia?

Surveying date:
School ID:
Classroom ID:
Student ID:

 NDIO HAPANA

10. Kama watu wangejua kwamba umewahi kupata hedhi, ungeogopa kwamba walimu wasingekuwa waelewa au wenye msaada?
     NDIO HAPANA

11. Kama watu wangejua kwamba umewahi kupata hedhi, ungeogopa kuhimizwa kuolewa au kuwa na mpenzi?
     NDIO HAPANA

12. Unakubaliana na kauli ifuatayo: "Nyumbani kwetu, wanawake/wasichana lazima waone aibu kuongelea au kuweka wazi hedhi yao (damu, maumivu, vifaa vya kujiweka safi) mbele ya wanafamilia."
     nakubaliana sana nakubaliana katikati sikubaliani sikubaliani kabisa

13. Unakubaliana na kauli ifuatayo: "Nyumbani kwetu, wanawake na wasichana ambao wako hedhini wanazuiliwa kufanya shughuli fulani."
     NDIO HAPANA Sijui

    Kama ulijibu NDIO, shughuli gani? Chagua zote zinazokubalika

    h) Kushika vyanzo vya maji au wanyama
    i) Kupika
    j) Kufua
    k) Mikutano ya hadhara kama vile mikutano ya kidini, mikutano ya jumuiya
    l) Kulala sehemu yake ya kila siku
    m) Kutumia choo ambacho huwa anatumia siku zote
    n) Nyingine: ______________________________

14. Unakubali kwamba wasichana lazima waolewe mara tu baada ya kuvunja ungo.
     nakubaliana sana nakubaliana katikati sikubaliani sikubaliani kabisa

15. Unakubali kawmba wasichana wanakuwa wakubwa kiasi cha kutosha kuanza kufanya mapenzi wakishavunja ungo
     nakubaliana sana nakubaliana katikati sikubaliani sikubaliani kabisa

Kipengele 7: Jibu sehemu hii kama tu umewahi kupata hedhi.

1. Umewahi kupata hedhi?

     NDIO (endelea) HAPANA (ruka hadi kipengele kinachofuata (Kipengele 7))

2. Wakati wa hedhi yako ya mwisho, je, kulikuwa na siku ambazo hukuweza kuja shuleni kwa sababu ya hedhi?

Surveying date:
School ID:
Classroom ID:
Student ID:

☐ NDIO ☐ HAPANA

Kama ndio ulibidi ukae nyumbani kwa siku ngapi kwa ajili ya hedhi? _____________

Kama ndio, kwanini ulikosa kuja shule? Tafadhali chagua yote yanayokubalika.

☐ maumivu ☐ kutokuwa na cha kutumia
☐ kuogopa nitavuja ☐ kuogopa harufu
☐ kuona aibu ☐ hakuna ba kubadilishia [vifaa vya usafi]
☐ kutoruhusiwa kushiriki ☐ kusikia kutapika
☐ kutojisikia raha ☐ nyingine ________

3. Wakati wa hedhi yako ya mwisho, uliwahi kuondoka shule mapema kwa sababu ya hedhi yako?

☐ NDIO ☐ HAPANA

Kama ndio, kwanini uliondoka mapema? Tafadhali chagua yote yanayokubalika.

☐ maumivu ☐ nilichoka
☐ kuhitaji kubadilishia [vifaa vya usafi] ☐ kuhitaji kunawa
☐ kuvuja ☐ kutaniwa
☐ nyingine ____

4. Wakati wa hedhi yako ya mwisho, je, **ulishiriki** darasani kama ambavyo huwa ni kawaida yako kushiriki (kipindi hauko hedhini)?

☐ NDIO ☐ HAPANA

Kama ndio, kwanini haukushiriki sana? Tafadhali chagua yote yanayokubalika.

☐ woga ☐ aibu
☐ maumivu ya tumbo na jumla ☐ vigumu kuwa makini
☐ kuogopa kusimama ☐ kuchoka
☐ nyingine _______________

5. Wakati wa hedhi yako ya mwisho, uliweza kuwa makini darasani kama ambavyo huwa ni kawaida yako kushiriki (kipindi hauko hedhini)?

☐ NDIO ☐ HAPANA

Kama hapana, kwanini haukuweza kuwa makini kama kawaida? Tafadhali chagua yote yanayokubalika.

☐ woga ☐ aibu
☐ maumivu ya tumbo na jumla ☐ vigumu kuwa makini
☐ kuogopa kusimama ☐ kuchoka
☐ nyingine _______________

6. Je, unaogopa kutaniwa kuhusu kuvuja damu ya hedhi shuleni?

☐ NDIO ☐ HAPANA

Kama ndio, na nani? Tafadhali chagua yote yanayokubalika.

☐ wasichana wa rika lako ☐ wavulana wa rika lako ☐ walimu
☐ hakuna mtu ☐ wengine ______

7. Unaogopa kutaniwa kuhusu harufu shuleni ukiwa hedhini?

☐ NDIO ☐ HAPANA

Surveying date:
School ID:
Classroom ID:
Student ID:

Kama ndio, nan ani? Tafadhali chagua yote yanayokubalika.
 wasichana wa rika lako wavulana wa rika lako walimu
 hakuna mtu wengine ______

8. Unaogopa kujibu maswali darasani ukiwa hedhini?
 NDIO HAPANA

9. Unafikiri baadhi ya wanafunzi wenzako wanaweza kutambua ukiwa hedhini?
 Ndio, mara nyingi Ndio, mara chache Hapana, kamwe

Kipengele 8: Kutaniwa

1. Je, umewahi kutaniwa kuhusu hedhi yako?
 NDIO HAPANA

2. Kutoka kwa wale marafiki wa KIUME uliowaandika juu (Kipengele 2, Swali 2), **wangapi** umewaona wakiwatania wasichana kuhusu hedhi zao?
 Hakuna Rafiki 1 Marafiki 2 Marafiki 3 Marafiki 4 Marafiki 5

3. Kutoka kwa wale marafiki wa KIKE uliowaandika juu (Kipengele 2, Swali 1), umeshawahi kujadili kuhusu hedhi ni **wangapi** kati yao?
 Hakuna Rafiki 1 Marafiki 2 Marafiki 3 Marafiki 4 Marafiki 5

4. Je, ni sawa kwa msichana kumuomba mpenzi wake kulipia bidhaa?
 NDIO HAPANA
Kama ndio, zipi?
____

Kipengele 9: Maswali juu ya Vitu Tunavyovijali

1. Walimu wangu hunitia moyo na kunipa msaada
 nakubali sana nakubali katikati sikubali sikubali sana

2. Elimu ni ya muhimu zaidi kwa wavulana kuliko wasichana
 nakubali sana nakubali katikati sikubali sikubali sana

3. Nina wasiwasi karibu muda wote
 nakubali sana nakubali katikati sikubali sikubali sana

4. Nimeridhika na Maisha yangu
 nakubali sana nakubali katikati sikubali sikubali sana

Kama hujapata hedhi, nenda Kipengele 10.

Surveying date:
School ID:
Classroom ID:
Student ID:

---

1. Najisikia aibu nikiwa hedhini
   ☐ nakubali sana ☐ nakubali ☐ katikati ☐ sikubali ☐ sikubali sana

2. Najisikia mwenye afya nikipata hedhi
   ☐ nakubali sana ☐ nakubali ☐ katikati ☐ sikubali ☐ sikubali sana

3. Najisikia kutengwa kutoka wengine nikiwa hedhini
   ☐ nakubali sana ☐ nakubali ☐ katikati ☐ sikubali ☐ sikubali sana

4. Najisikia wasiwasi nikiwa hedhini
   ☐ nakubali sana ☐ nakubali ☐ katikati ☐ sikubali ☐ sikubali sana

5. Naogopa kwamba nanuka nikiwa hedhini
   ☐ nakubali sana ☐ nakubali ☐ katikati ☐ sikubali ☐ sikubali sana

6. Najisikia jasiri zaidi nikitumia pedi nikiwa hedhini
   ☐ nakubali sana ☐ nakubali ☐ katikati ☐ sikubali ☐ sikubali sana

Kipengele 10: Maswali ya Bei na WTP

1. Tutakuuliza maswali kadhaa ambapo unaweza ukachagua kutoka vitu viwili. Tazama kila swali kama linajitegemea. Jibu kila swali kiukweli kwa sababu unaweza kujishindia chaguo lako.
   a. Ungependelea …?
      ☐ Pedi ya kutumia zaidi ya mara moja AU ☐ TSH 0

   b. Ungependelea …?
      ☐ Pedi ya kutumia zaidi ya mara moja AU ☐ TSH 1500

   c. Ungependelea …?
      ☐ Pedi ya kutumia zaidi ya mara moja AU ☐ TSH 3000

   d. Ungependelea …?
      ☐ Pedi ya kutumia zaidi ya mara moja AU ☐ TSH 4500

   e. Ungependelea …?
      ☐ Pedi ya kutumia zaidi ya mara moja AU ☐ TSH 6000

   f. Ungependelea …?
      ☐ Pedi ya kutumia zaidi ya mara moja AU ☐ TSH 7500

   g. Ungependelea …?
      ☐ Pedi ya kutumia zaidi ya mara moja AU ☐ TSH 9000

   h. Ungependelea …?
      ☐ Pedi ya kutumia zaidi ya mara moja AU ☐ TSH 10,000

Surveying date:
School ID:
Classroom ID:
Student ID:

---

i. Ungependelea …?
☐ Pedi ya kutumia zaidi ya mara moja AU ☐ TSH 11,500

j. Ungependelea …?
☐ Pedi ya kutumia zaidi ya mara moja AU ☐ TSH 13,000

Kipengele 11: Demografia

1. Jina lako nani? (la kwanza na la pili)______________________________
2. Umeishi muda gani katika makazi uliyopo? ____________________
3. Umezaliwa mwezi gani na mwaka gani?______________________________
4. Ulifikisha miaka mingapi kwenye tarehe yako ya kuzaliwa iliyopita? ______________________________
5. Upo darasa la ngapi?______________________________
6. Ulianza shul ukiwa na umri gani? ______________________________
7. Tafadhali chagua kabila lako linalokutambulisha
   ☐ Mchagga
   ☐ Mmasai
   ☐ Msukuma
   ☐ Mhaya
   ☐ Msumbwa
   ☐ kabila linginelo:______________________________
8. Tafadhali chagua dhehebu linalokutambulisha
   ☐ Mkristo
   ☐ Muislamu
   ☐ Dhehebu jinginelo:______________________________
   ☐ Sina dhehebu
9. Unaishi na nani kwa sasa?
   i. ☐ wazazi ☐ bibi na babu ☐ peke yako
   ii. ☐ ndugu uliozaliwa nao ☐ wanafamilia wengine ☐ bweni la shule
   iii. ☐ bweni/hosteli binafsi ☐ nyingine ______________________________
10. Unaweza kuoga nyumbani?
    iv. ☐ NDIO ☐ HAPANA
11. Unatumia jumla ya kiasi gani cha pesa kwa hedhi? ____________
12. Chanzo chako kikuu cha pesa ni kipi? Tafadhali chagua moja.

Surveying date:
School ID:
Classroom ID:
Student ID:

---

 mama baba mpenzi
 dada / kaka nyingine kazi
 ufadhili

13. Huwa unaitumia [hela] kufanya nini? Tafadhali chagua matumizi yako makuu matatu.
 nguo
 usafi binafsi
 vipodozi
 simu ya mkononi
 pedi
 nywele
 vifaa vya shule
 kitabu
 usafiri
 nyingine

14. Unatumia kiasi gani cha pesa kwenye pedi kila hedhi? ____________________________

15. Umekosa shule ndani ya siku 7 zilizopita?
 NDIO HAPANA

Kama Ndio, sababu ilikuwa nini? ____

SUPPLEMENTARY MATIERAL
QUESTIONNAIRE for "Period teasing, stigma and knowledge: a survey of adolescent boys and girls in Northern Tanzania" by Benshaul-Tolonen et al. 2020

Surveying date:
School ID:
Classroom ID:
Student ID:

BOYS QUESTIONNAIRE

Questionnaire

Section 1: Friends

1. We would now like you to tell us about the 5 closest **MALE** friends you have in the school. Please mention the names of your closest friends. Only list friends who go to this school, and who are enrolled in the same year as you, and that you count as friends.

Write the full name and class of the friends mentioned

a. ______________________________
b. ______________________________
c. ______________________________
d. ______________________________
e. ______________________________

2. In your opinion, who are the most popular boys in school?

Write the full name and class of the boys mentioned

a. ______________________________
b. ______________________________
c. ______________________________
d. ______________________________
e. ______________________________

Section 2: Information
We would now like to ask you some questions about periods.

1. Why do a girl or a woman get her period? Select all that apply
   a. ☐ personal sin
   b. ☐ due to outside evil forces
   c. ☐ because she did not become pregnant that month
   d. ☐ release of bad blood
   e. ☐ other
   f. ☐ I do not know

2. Approximately how often does a girl (who have ever had her period) get her period? (Select one)
   ☐ once in her life
   ☐ once every year
   ☐ every week
   ☐ every month

3. How long does a menstrual period last on average? (Select one)
   ☐ one hour

Surveying date:
School ID:
Classroom ID:
Student ID:

---

 one day
 a few days
 two weeks
 all the time

4. At what age do girls generally get their period for the first time?

____

5. At what age do women stop getting their period?

____

6. Select the correct answer.
Please select Yes or No to indicate if the following statements are true.

h) When a girl reaches menarches (her first menstruation), it means she could get pregnant if she has sex.
 Yes No I do not know
i) Menstruation blood is the shedding of the endometrium lining
 Yes No I do not know
j) Menstrual period indicates a woman is not pregnant
 Yes No I do not know
k) Ovulation happens on average 14 days before period
 Yes No I do not know
l) Ovulation happens during period
 Yes No I do not know
m) Is it common for girls to have physical discomfort when they have their period?
 Yes No I do not know
n) Do girls experience the following on their period?
Bad mood Yes No I do not know
Stress Yes No I do not know
Fatigue Yes No I do not know

Section 3: Personal Experience

1. Has anyone told you about girls' periods and why it is happening?
 YES NO

If so, who? Please select one.
 mother father
 grandmother grandfather
 sister brother
 female friend male friend

Surveying date:
School ID:
Classroom ID:
Student ID:

☐ aunt or other female family member	☐ uncle or male family member
☐ health worker	☐ priest / religious leader
☐ female teacher	☐ male teacher
☐ girlfriend/partner	☐ other

2. Did you ever receive information about menstruation from the following:

e)	Internet	☐ YES	☐ NO
f)	Health worker	☐ YES	☐ NO
g)	School curricula	☐ YES	☐ NO
h)	Informational pamphlet (ads, billboards)	☐ YES	☐ NO

3. What was your reaction the first time that you learned about periods? Were you…
(Please select one from each group 5, where 3 is neutral.)

	**1**	**2**	**3**	**4**	**5**	
surprised	☐	☐	☐	☐	☐	not at all surprised
afraid	☐	☐	☐	☐	☐	not at all afraid
excited	☐	☐	☐	☐	☐	not at all excited
confused	☐	☐	☐	☐	☐	not at all confused
confident	☐	☐	☐	☐	☐	not at all confident
disgusted	☐	☐	☐	☐	☐	not at all disgusted
proud	☐	☐	☐	☐	☐	not at all proud
ashamed	☐	☐	☐	☐	☐	not at all ashamed

Section 4: Peers' Period Management

1. Please estimate the share of female classmates who have received their periods. You will receive 1 USD if you guess the right answer.

☐ 0%

SUPPLEMENTARY MATIERAL
QUESTIONNAIRE for “Period teasing, stigma and knowledge: a survey of adolescent boys and girls in Northern Tanzania” by Benshaul-Tolonen et al. 2020

Surveying date:
School ID:
Classroom ID:
Student ID:

☐ 1-25%
☐ 26-50%
☐ 51-75%
☐ 76-100%

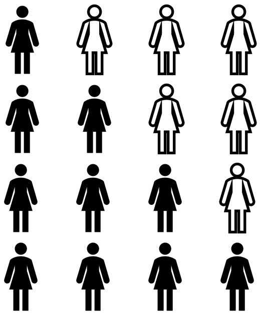

2. Sometimes girls are teased about their periods. Please estimate the share of female classmates who have been teased about their periods. You will receive 1 USD if you guess the right answer.

☐ 0%
☐ 1-25%
☐ 26-50%
☐ 51-75%
☐ 76-100%

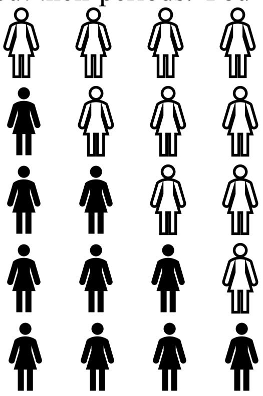

3. Please estimate **the most common** method of menstrual sanitation among your peers in the class. Please select only one.

☐ cloth ☐ pads
☐ tampons ☐ natural products (e.g. grass, leaves, cow dung)
☐ mattress stuffing ☐ menstrual cup
☐ nothing ☐ other ________________________________

3. How confident are you that you know what your female classmates use when they are menstruating?
   Not at all confident 1 2 3 4 5 Very confident

4. If they use sanitary pads, do you know how your female peers dispose of them?
   ☐ YES ☐ NO

5. If you answered yes to the last question above, what is the most common thing they do?
   ☐ They don’t dispose of them (they use it over and over)
   ☐ dust bin
   ☐ toilet/latrine
   ☐ burn them
   ☐ bury them
   ☐ other ________________________________

Surveying date:
School ID:
Classroom ID:
Student ID:

Section 5: Menstruation Attitudes

1. Do you feel safe in the latrines at school?
   □ YES □ NO

2. Do you know when your female classmates have their period?
   □ YES □ NO

3. Do you think that girls would feel ashamed if you knew they were on their period?
   □ YES □ NO

4. Do you feel like periods are something to hide at school?
   □ YES □ NO

5. Do you think that girls afraid of telling people about their period?
   □ YES □ NO

6. Is it appropriate for girls to talk about their periods in public?
   □ Very appropriate □ Appropriate □ Neutral □ Inappropriate □ Very inappropriate

7. What would happen if a girl spoke about her period in public?
   ____

8. Sometimes it matters <u>who</u> girls talks to about their periods.

   a. Is it appropriate for girls to talk about their periods with their female classmates?
   □ Very appropriate □ Appropriate □ Neutral □ Inappropriate □ Very inappropriate

   b. Is it appropriate for girls to talk about their periods with their male classmates?
   □ Very appropriate □ Appropriate □ Neutral □ Inappropriate □ Very inappropriate

   c. Is it appropriate for girls to talk about their periods with their female teacher?
   □ Very appropriate □ Appropriate □ Neutral □ Inappropriate □ Very inappropriate

   d. Is it appropriate for girls to about their periods with their male teacher?
   □ Very appropriate □ Appropriate □ Neutral □ Inappropriate □ Very inappropriate

   e. Is it appropriate for girls to about their periods with their father?
   □ Very appropriate □ Appropriate □ Neutral □ Inappropriate □ Very inappropriate

   f. Is it appropriate for girls to about their periods with their mother?
   □ Very appropriate □ Appropriate □ Neutral □ Inappropriate □ Very inappropriate

9. Do you agree with the following statement: "In my house, women/girls should be ashamed to talk about or reveal their period (blood, pains, sanitary materials) in front of my family members."

Surveying date:
School ID:
Classroom ID:
Student ID:

---

 strongly agree agree neutral disagree strongly disagree

10. Do you agree with the following statement: "In my house, women and girls who are menstruating are restricted from certain activities."
     YES NO Don't know

If you answered YES, what activities? Select all that apply

o) Touching water sources or animals
p) Cooking
q) Washing dishes
r) Public gatherings like religious gatherings, community meetings
s) Sleeping in her normal spot
t) Using the normal toilet/latrine
u) Other: ______________________________

11. Would you like to have someone that you can ask about periods?
     YES NO

Section 6: Teasing

1. From the MALE friends you wrote above (Section 1, Question 1), **how many** have you seen teasing girls about their periods?
    No friends 1 friend 2 friends 3 friends 4 friends 5 friends

2. From the friends mentioned above, with how many have you ever discussed periods?
    No friends 1 friend 2 friends 3 friends 4 friends 5 friends

3. Do your close friends tease girls about their period? (if Never/Don't know, skip to Question 6)
    Always Often Sometimes Never Don't know

4. If so, when does this happen?
    When they smell bad
    When their clothes get blood stains
    When they find pads or other products in girls' belongings
    When girls don't want to participate in sports or class because of their period
    When they think that a girl has her period

    Other: ______________________________

5. Why do you think that your friends tease girls about their periods?
    Because periods are embarrassing
    Because periods are unnatural
    Because it is due to outside evil forces

Surveying date:
School ID:
Classroom ID:
Student ID:

□ Because periods are a personal sin
□ Because boys are immature, or they don't understand periods

□ Other: ____

6. Have you ever teased a girl about their period?
   □ YES □ NO

7. If YES, when? Check all that apply.
   □ When they smell bad
   □ When their clothes get blood stains
   □ When I find pads or other products in girls' belongings
   □ When girls don't want to participate in sports or class because of their period
   □ When I think that a girl has her period

   □ Other: ______________________________

8. If YES why did you tease them?
   □ Because periods are embarrassing
   □ Because periods are unnatural
   □ Because it is due to outside evil forces
   □ Because periods are a personal sin
   □ Because other boys were also teasing

   □ Other: ____

9. When did you last tease a girl because of her period?
   □ In the last week
   □ In the last month
   □ In the last 6 months
   □ In the last year
   □ Over 1 year ago

10. When was the last time you saw a **classmate** tease a girl about their period?
    □ In the last week
    □ In the last month
    □ In the last 6 months
    □ In the last year
    □ Over 1 year ago

11. What do you do if you see a classmate tease a girl about their period?
    □ Nothing
    □ I talk with the classmate who teased in private
    □ I talk with the girl who was teased in private

Surveying date:
School ID:
Classroom ID:
Student ID:

 I tell the classmate to stop
 I also tease the girl
 I tell the teacher or another adult

 Other ______________________

Section 7: Partners

1. Have you ever had a girlfriend?
    YES NO

2. Do you have a girlfriend right now?
    YES NO (go to question 31)

3. How old is your girlfriend?
____

4. Is it okay for a girl to ask her boyfriend to pay for items?
    YES NO

   30a. If so, what? (select all that apply)
    clothes food pads other ______

5. Is it okay for boys to expect girls to have sex with them after giving them gifts or paying for items?
    YES NO

6. Do you agree that girls should get married as soon as they get their period.
 strongly agree agree neutral disagree strongly disagree

7. Do you agree that girls are old enough to have sex after they get their period
 strongly agree agree neutral disagree strongly disagree

Section 8: Value Questions

1. My parents/caretakers encourage me to do well in school
    strongly agree agree neutral disagree strongly disagree

2. My teachers give me a lot of encouragement and support
    strongly agree agree neutral disagree strongly disagree

3. Education is more important for boys than for girls

Surveying date:
School ID:
Classroom ID:
Student ID:

---

 strongly agree agree neutral disagree strongly disagree

4. Things in the future will work out OK for me
 strongly agree agree neutral disagree strongly disagree

5. I am worried nearly all the time
 strongly agree agree neutral disagree strongly disagree

6. I am satisfied with my life
 strongly agree agree neutral disagree strongly disagree

Section 9: Demographics

1. What is your name? (first and last) ____
2. How long have you been living continuously in (place of residence)? _______________________
3. In what month and year where you born?____
4. How old were you at your last birthday? ____
5. What grade are you in?____
6. At what age did you start school? ____
7. Who do you currently live with?
 parents grandparents alone
 siblings other family members boarding school
 boarding house/hostel other ____________________________
8. Can you wash your body at home?
 YES NO
9. How much money do you spend per month total? ___________ (order changed)
10. What is your main source of money? Please select one.
 mother father girlfriend/boyfriend
 sister /brother other work
 scholarship
11. What do you spend it on? Please select the top three expenses.
 clothes

Surveying date:
School ID:
Classroom ID:
Student ID:

---

 personal hygiene
 make up
 mobile phone
 hair
 school products
 food
 transport
 other

12. Did you miss school in the past 7 days?
 YES NO

If Yes, what was the reason? ____

GIRLS QUESTIONNAIRE

Questionnaire

Section 1: Friends

1. We would now like you to tell us about the 5 closest **FEMALE** friends you have in the school. Please mention the names of your closest friends. Only list friends who go to this school, and who are enrolled in the same year as you, and that you count as friends.

Write the full name and class of the friends mentioned

a. ________________________________
b. ________________________________
c. ________________________________
d. ________________________________
e. ________________________________

2. We would now like you to tell us about the 5 closest **MALE** friends you have in the school. Please mention the names of your closest friends. Only list friends who go to this school, and who are enrolled in the same year as you, and that you count as friends.

Write the full name and class of the friends mentioned

a. ________________________________
b. ________________________________
c. ________________________________
d. ________________________________
e. ________________________________

Section 2: Information

SUPPLEMENTARY MATIERAL
QUESTIONNAIRE for "Period teasing, stigma and knowledge: a survey of adolescent boys and girls in Northern Tanzania" by Benshaul-Tolonen et al. 2020

Surveying date:
School ID:
Classroom ID:
Student ID:

We would now like to ask you some questions about periods.

1. Why do a girl or a woman get her period?. Select all that apply.
   - personal sin
   - due to outside evil forces
   - because she did not become pregnant that month
   - release of bad blood
   - other
   - I do not know

2. Approximately, how often does a girl (who have ever had her period) get her period? Select one.
   - once in her life
   - once every year
   - every week
   - every month
   - I do not know

3. How long does a menstrual period last on average? Select one.
   - one hour
   - one day
   - a few days
   - two weeks
   - all the time
   - I do not know

4. At what age do girls generally get their period for the first time?

   ____

5. At what age do women stop getting their period?

   ____

6. Select the correct answer
   Please select Yes or No to indicate if the following statements are true.

   h) When a girl reaches menarches (her first menstruation), it means she could get pregnant if she has sex.
   Yes   No   I do not know
   i) Menstruation blood is the shedding of the endometrium lining
   Yes   No   I do not know
   j) Menstrual period indicates a woman is not pregnant
   Yes   No   I do not know
   k) Ovulation happens on average 14 days before period
   Yes   No   I do not know
   l) Ovulation happens during period

Surveying date:
School ID:
Classroom ID:
Student ID:

☐ Yes ☐ No ☐ I do not know

m) Is it common for girls to have physical discomfort when they have their period?

☐ Yes ☐ No ☐ I do not know

n) Do most girls experience the following on their period?

Bad mood	☐ Yes	☐ No	☐ I do not know
Stress	☐ Yes	☐ No	☐ I do not know
Fatigue	☐ Yes	☐ No	☐ I do not know

Section 3: Personal Experience

1. Has anyone told you about girls' periods and why it is happening?

   ☐ YES ☐ NO

   If so, who? Please select all that apply.

☐ mother	☐ father
☐ grandmother	☐ grandfather
☐ sister	☐ brother
☐ female friend	☐ male friend
☐ aunt or other female family member	☐ uncle or male family member
☐ health worker	☐ priest / religious leader
☐ female teacher	☐ male teacher
☐ boyfriend/partner	☐ other

2. Did you ever receive information about menstruation from the following:

a. Internet	☐ YES	☐ NO
b. Health worker	☐ YES	☐ NO
c. School materials (books, classes, homework, etc.)	☐ YES	☐ NO
d. Informational pamphlet (ads, billboards)	☐ YES	☐ NO

3. What was your reaction the first time that you learned about periods? Were you…
(Please select one from each group 5, where 3 is neutral.)

	**1**	**2**	**3**	**4**	**5**	
surprised	☐	☐	☐	☐	☐	not at all surprised
afraid	☐	☐	☐	☐	☐	not at all afraid
excited	☐	☐	☐	☐	☐	not at all excited
confused	☐	☐	☐	☐	☐	not at all confused

Surveying date:
School ID:
Classroom ID:
Student ID:

---

confident	☐	☐	☐	☐	☐	not at all confident
disgusted	☐	☐	☐	☐	☐	not at all disgusted
proud	☐	☐	☐	☐	☐	not at all proud
ashamed	☐	☐	☐	☐	☐	not at all ashamed

4. Has anyone told you what you should do when you start menstruating?
   ☐ YES ☐ NO

   If so, what advice did you receive? Please select all that apply.
   ☐ how to use towels/fabrics/linen
   ☐ how to stay clean/wash
   ☐ how to manage pain
   ☐ things/activities to avoid during menstruation
   ☐ other ___________

5. Check all the people that you would **WANT** to talk to about your period.

☐ mother	☐ father
☐ grandmother	☐ grandfather
☐ sister	☐ brother
☐ female friend	☐ male friend
☐ aunt or other female family member	☐ uncle or male family member
☐ health worker	☐ priest / religious leader
☐ female teacher	☐ male teacher
☐ boyfriend/partner	☐ other
☐ no one	

6. With whom would you **ABSOLUTELY NOT** want to talk to about your period. Please select all that apply.

☐ mother	☐ father
☐ grandmother	☐ grandfather
☐ sister	☐ brother
☐ female friend	☐ male friend
☐ aunt or other female family member	☐ uncle or male family member
☐ health worker	☐ priest / religious leader
☐ female teacher	☐ male teacher
☐ boyfriend/partner	☐ other
☐ no one	

Surveying date:
School ID:
Classroom ID:
Student ID:

---

7. Have you had your period?
   ☐ Yes **(continue to next question)** ☐ No **(go to Section 4, Question 1)**

8. At what age did you have your first period? _______

9. Was the first time that you got your period a positive, negative, or neutral experience?
   ☐ positive ☐ negative ☐ neutral

10. What was your reaction the first time that you got your period? Were you…
(Please select one from each group 5, where 3 is neutral.)

	**1**	**2**	**3**	
surprised	☐	☐	☐	not at all surprised
afraid	☐	☐	☐	not at all afraid
excited	☐	☐	☐	not at all excited
confused	☐	☐	☐	not at all confused
confident	☐	☐	☐	not at all confident
disgusted	☐	☐	☐	not at all disgusted
proud	☐	☐	☐	not at all proud
ashamed	☐	☐	☐	not at all ashamed

11. Think back to the time before you got your period. Did anyone tell you about your period before it started?
    ☐ YES ☐ NO

If so, who? Please select all that apply.

If so, who? Please select all that apply.

☐ mother ☐ father
☐ grandmother ☐ grandfather
☐ sister ☐ brother
☐ female friend ☐ male friend
☐ aunt or other female family member ☐ uncle or male family member

Surveying date:
School ID:
Classroom ID:
Student ID:

☐ health worker	☐ priest / religious leader
☐ female teacher	☐ male teacher
☐ boyfriend/partner	☐ other

12. How many days does your period last?
    __________days

13. From a scale of 1 to 5 where 1 is no pain and 5 very strong pain; How much physical pain do you experience when you have your period?

	1	2	3	4	5	
No pain at all	☐	☐	☐	☐	☐	*Very strong pain*

14. On a scale from 1 to 5, where 1 is less pain and 5 is more pain and 3 is the same; How do you think that the pain you feel during your period is compared to the pain that your peers experience?

	1	2	3	4	5	
I experience much less pain	☐	☐	☐	☐	☐	*I experience much stronger pain*

15. From a scale of 1 to 5 where 1 is very light bleeding and 5 very heavy bleeding; How much do you bleed when you have your period?

	1	2	3	4	5	
very light bleeding	☐	☐	☐	☐	☐	*Very heavy bleeding*

16. On a scale from 1 to 5, where 1 is lighter bleeding and 5 is heavier bleeding and 3 is the same; Do you think that you bleed more, less, or the same as your peers?

	1	2	3	4	5	
I have much lighter bleeding	☐	☐	☐	☐	☐	*I have much heavier bleeding*

17. A menstrual cycle lasts from the first day of period (bleeding) until the day before the beginning of the next period (first day of bleeding). Do you know how long your menstrual cycle is on average? In other word, do you keep track of how often do you bleed?
    ☐ YES ☐ NO

18. Do you think that for you the time between bleedings varies a lot?
    ☐ YES ☐ NO

19. Do you find it hard to know when you are going to bleed soon and be prepared?
    ☐ YES ☐ NO

20. During your period, or immediately before, do you ever have…:

Bad mood?	☐ YES	☐ NO
Stress or anxiety?	☐ YES	☐ NO
Fatigue?	☐ YES	☐ NO

21. Do you use painkillers (like for example aspirin) to manage pain when you have your period?
    ☐ Always ☐ Sometimes ☐ Rarely ☐ Never

Surveying date:
School ID:
Classroom ID:
Student ID:

BREAK HERE FOR SECTION 4 WITH AFRIPADS

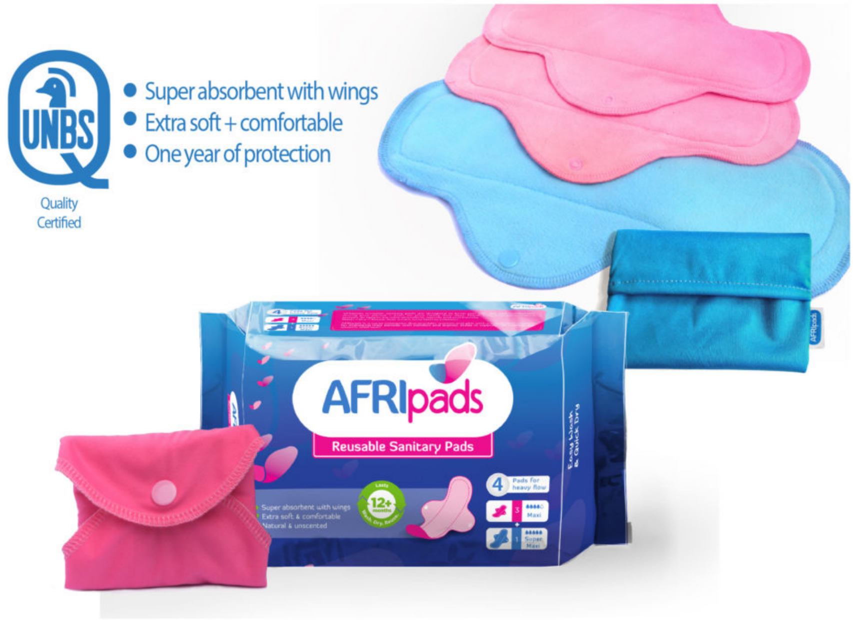

Pedi za kusafisha zinazotumika zaidi ya mara moja ni kitambaa kinachovaliwa kwenye chupi (nguo ya ndani) kuzuia damu ya hedhi kuvujia kwenye nguo. Inaweza kuoshwa, kukaushwa na kutumiwa kwa mwaka 1. Picha hapo juu inaonesha Afripads, aina mojawapo ya pedi za kutumiwa zaidi ya mara moja. Kila pakiti ina pedi 4.

1. *Je umewahi Kuona ama Kusikia kuhusu pedi za kusafisha zinazotumika Zaidi ya mara moja?*

   ☐ *Ndio* ☐ *Hapana*

2. *Je umewahi Kutumia pedi za kusafisha zinazotumika Zaidi ya mara moja?*

   ☐ *Ndio* ☐ *Hapana*

3. What do you **sometimes** use when you are menstruating? Please select **all that apply.**
   ☐ cloth ☐ pads
   ☐ tampons ☐ natural materials (grass, leaves, cow dung, etc.)
   ☐ mattress stuffing ☐ menstrual cup
   ☐ nothing ☐ other ________

4. What do you **most often** use when you are menstruating? Please select **only one**.
   ☐ cloth ☐ pads
   ☐ tampons ☐ natural materials (grass, leaves, cow dung, etc.)
   ☐ mattress stuffing ☐ menstrual cup
   ☐ nothing ☐ other ________

Surveying date:
School ID:
Classroom ID:
Student ID:

---

5. Do you ever use cloth?
   ☐ YES ☐ NO
   a. If you ever use a cloth, do you wash the fabrics you use during menstruation with soap?
   ☐ YES ☐ NO ☐ I don’t use a cloth
   b. If you ever use a cloth, do you dry the fabrics you have washed in the sun?
   ☐ YES ☐ NO ☐ I don’t use a cloth
   If no, where ____________________

6. What is your preferred method of menstrual sanitation? Please **select one.**
   ☐ cloth ☐ pads
   ☐ tampons ☐ natural products (grass, leaves, cow dung, etc.)
   ☐ mattress stuffing ☐ menstrual cup
   ☐ nothing ☐ other ____________________

7. Do you always use your preferred method?
   ☐ YES ☐ NO

8. Think of a month when you did not use your preferred method. Why did you **NOT** use your preferred method?
   ☐ not enough money to buy ☐ the method wasn’t available
   ☐ no parental permission ☐ I didn’t know how to
   ☐ I was embarrassed ☐ the method wasn’t safe
   ☐ other ____________________

9. How often do you change sanitation during your period each day? __________

10. How do you dispose of your sanitary product (**the one that you chose in question 24**)?
   ☐ I don’t (I use it over and over) ☐ dust bin
   ☐ toilet/latrine ☐ burn it
   ☐ bury it ☐ other ____________________

Section 4: Peers’ Period Management (UPDATED TO SECTION 5)

1. Please estimate the share of female classmates who have received their periods.

   ☐ 0% 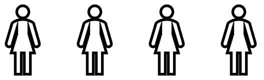

   ☐ 1-25% 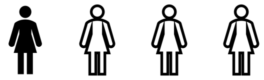

   ☐ 26-50% 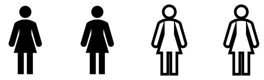

   ☐ 51-75% 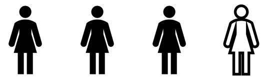

☐ 76-100% 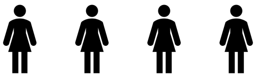

2. Sometimes girls are teased about their periods. Please estimate the share of female classmates who have been teased about their periods. .

☐ 0% 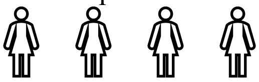

☐ 1-25% 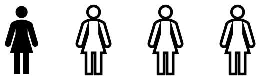

☐ 26-50% 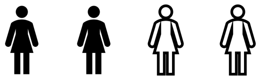

☐ 51-75% 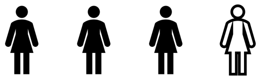

☐ 76-100% 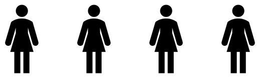

3. Please estimate **the most common** method of menstrual sanitation among your peers in the class. Please select only one.

☐ cloth	☐ pads
☐ tampons	☐ natural materials (grass, leaves, cow dung, etc.)
☐ mattress stuffing	☐ menstrual cup
☐ nothing	☐ other _________

4. How confident are you that you know what your female classmates use when they are menstruating?

Not at all confident ☐1 ☐2 ☐3 ☐4 ☐5 *Very confident*

5. If they use sanitary pads, do you know how your female peers dispose of them?

☐ YES ☐ NO

6. If you answered yes to the question above, what is the most common thing they do?

☐ They don’t dispose of them (they use it over and over)
☐ dust bin
☐ toilet/latrine
☐ burn them
☐ bury them
☐ other ____

Section 6: Management at School

1. Do you feel safe in the latrines at school?

☐ YES ☐ NO

2. Do you feel safe in the latrines at school when you have your period?

Surveying date:
School ID:
Classroom ID:
Student ID:

☐ YES ☐ NO

3. Do you know when your female classmates have their period?
   ☐ YES ☐ NO

4. Do you feel like having period is something that one must hide from others?
   ☐ YES ☐ NO

5. Would you feel ashamed if other girls knew that you were on your period?
   ☐ YES ☐ NO

6. Would you feel ashamed if boys knew that you were on your period?
   ☐ YES ☐ NO

7. If people knew that you have ever received your period, would you be afraid of teasing?
   ☐ YES ☐ NO
   If so, by whom? Please select all that apply.
   ☐ female peers ☐ male peers ☐ teachers ☐ no one ☐ other______

8. If people knew that you have ever received your period, would you be afraid of being touched against your will, or asked insistently to go out with someone?
   ☐ YES ☐ NO
   If so, by whom? Please select all that apply.
   ☐ peers ☐ teachers ☐ family members ☐ no one ☐ other______

   If so, where are you afraid that this would occur?
   ☐ public bathrooms
   ☐ school bathrooms
   ☐ class room
   ☐ school yard
   ☐ on my way to or from school
   ☐ public space (such as a square)
   ☐ home
   ☐ nowhere
   ☐ other __________________________

9. If people knew that you have your period, would you be afraid of unwanted pregnancies or dishonor?
   ☐ YES ☐ NO

10. If people knew that you have ever received your period, would you be afraid of teachers not being understanding or helpful?
    ☐ YES ☐ NO

11. If people knew that you have ever received your period, would you afraid of the pressure to marry or take a boyfriend?
    ☐ YES ☐ NO

Surveying date:
School ID:
Classroom ID:
Student ID:

---

12. Do you agree with the following statement: "In my house, women/girls should be ashamed to talk about or reveal their period (blood, pains, sanitary materials) in front of my family members."
    - strongly agree - agree - neutral - disagree - strongly disagree

13. Do you agree with the following statement: "In my house, women and girls who are menstruating are restricted from certain activities."
    - YES - NO - Don't know

    If you answered YES, what activities? Select all that apply

    v) Touching water sources or animals
    w) Cooking
    x) Washing dishes
    y) Public gatherings like religious gatherings, community meetings
    z) Sleeping in her normal spot
    aa) Using the normal toilet/latrine
    bb) Other: ______________________________

14. Do you agree that girls should get married as soon as they get their period.
    - strongly agree - agree - neutral - disagree - strongly disagree

15. Do you agree that girls are old enough to have sex after they get their period
    - strongly agree - agree - neutral - disagree - strongly disagree

Section 7: Only fill this in if you ever had your period.

1. Did you ever have a period?

   - YES (continue) - NO (skip to next section (Section 7)) (NOW SECTION 8)

2. During your last period, were there days when you could not come to school because of your period?
   - YES - NO

   If yes how many days did you have to stay at home because of your period? _____________

   If yes, why did you miss school? Please select all that apply.
   - pain
   - nothing to use
   - afraid I will leak
   - afraid of odor
   - ashamed
   - no place to change
   - not allowed to take part
   - feel sick
   - don't feel comfortable
   - other ________

3. During your last period, did you ever leave school early because of your period?

Surveying date:
School ID:
Classroom ID:
Student ID:

 YES NO

If yes, why did you leave early? Please select all that apply.

 pain tired
 needed to change needed to wash
 leaked teased
 other ____

4. During your last period, did you **participate** as much in class as you normally do (when you do not have your period)?
    YES NO

   If no, why did you not participate as much? Please select all that apply.

    fear shame
    cramps and pain hard to concentrate
    afraid to stand up tired
    other ________________

5. During your last period, did you **concentrate** as much in class as you normally do (when you do not have your period)?
    YES NO

   If no, why did you not concentrate as much? Please select all that apply.

    fear shame
    cramps and pain hard to concentrate
    afraid to stand up tired
    other ________________

6. Are you afraid of being teased about leaking menstrual blood at school?
    YES NO

   If so, by whom? Please select all that apply.

    female peers male peers teachers no one other______

7. Are you afraid of being teased about odor at school when you have your period?
    YES NO

   If so, by whom? Please select all that apply.

    female peers male peers teachers no one other______

8. Are you afraid of answering a question in the classroom when you have your period?
    YES NO

9. Do you think that some of your classmates can tell when you have your period?
    Yes, most of the time Yes, only sometimes No, never

Section 8: Teasing

1. Have you ever been teased about your period?

Surveying date:
School ID:
Classroom ID:
Student ID:

 YES NO

2. From the MALE friends you wrote above (Section 2, Question 2), **how many** have you seen teasing girls about their periods?
    No friend 1 friend 2 friends 3 friends 4 friends 5 friends

3. From the FEMALE friends you wrote above (Section 2, Question 1), with **how many** have you ever discussed periods?
    No friend 1 friend 2 friends 3 friends 4 friends 5 friends

4. Is it okay for a girl to ask her boyfriend to pay for items?
    YES NO
   If so, what?
   ____

Section 9: Value Questions

1. My parents/caretakers encourage me to do well in school
    strongly agree agree neutral disagree strongly disagree

2. My teachers give me a lot of encouragement and support
    strongly agree agree neutral disagree strongly disagree

3. Education is more important for boys than for girls
    strongly agree agree neutral disagree strongly disagree

4. Things in the future will work out OK for me
    strongly agree agree neutral disagree strongly disagree

5. I am worried nearly all the time
    strongly agree agree neutral disagree strongly disagree

6. I am satisfied with my life
    strongly agree agree neutral disagree strongly disagree

If you have NOT had your period, go to Section 9.

7. I feel ashamed during my period
    strongly agree agree neutral disagree strongly disagree

8. I feel healthy when I have my period
    strongly agree agree neutral disagree strongly disagree

9. I feel isolated from others during my period

Surveying date:
School ID:
Classroom ID:
Student ID:

---

 strongly agree agree neutral disagree strongly disagree

10. I feel worried during my period
     strongly agree agree neutral disagree strongly disagree

11. I worry that I smell when I have my period
     strongly agree agree neutral disagree strongly disagree

12. I feel more confident during my period when using pads
     strongly agree agree neutral disagree strongly disagree

Section 10: Price Questions and WTP

1. We will ask you a few questions where you can choose between two items. Treat each question as a separate question. Answer each question truthfully because you might win your choice.
    a. Would you prefer…?
     A reusable sanitary pad OR TSH 0

    b. Would you prefer…?
     A reusable sanitary pad OR TSH 1500

    c. Would you prefer…?
     A reusable sanitary pad OR TSH 3000

    d. Would you prefer…?
     A reusable sanitary pad OR TSH 4500

    e. Would you prefer…?
     A reusable sanitary pad OR TSH 6000

    f. Would you prefer…?
     A reusable sanitary pad OR TSH 7500

    g. Would you prefer…?
     A reusable sanitary pad OR TSH 9000

    h. Would you prefer…?
     A reusable sanitary pad OR TSH 10,000

    i. Would you prefer…?
     A reusable sanitary pad OR TSH 11,500

    j. Would you prefer…?
     A reusable sanitary pad OR TSH 13,000

Section 11: Demographics

SUPPLEMENTARY MATIERAL
QUESTIONNAIRE for "Period teasing, stigma and knowledge: a survey of adolescent boys and girls in Northern Tanzania" by Benshaul-Tolonen et al. 2020

Surveying date:
School ID:
Classroom ID:
Student ID:

---

1. What is your name? (first and last) ______________________________

2. How long have you been living continuously in (place of residence)? ______________

3. In what month and year where you born?______________________________

4. How old were you at your last birthday? ______________________________

5. What grade are you in?______________________________

6. At what age did you start school? ______________________________

7. Please select the ethnicity that best describes you:

   ☐ Chagga
   ☐ Masai
   ☐ Sukuma
   ☐ Wahaya
   ☐ Wasumbwa
   ☐ Other :______________________

8. Please select the religion that best describes you:

   ☐ Christian
   ☐ Muslim
   ☐ Chagga
   ☐ Other :______________________
   ☐ No Religion

9. Who do you currently live with?
   i. ☐ parents ☐ grandparents ☐ alone
   ii. ☐ siblings ☐ other family members ☐ boarding school
   iii. ☐ boarding house/hostel ☐ other ________________

10. Can you wash your body at home?
   i. ☐ YES ☐ NO

11. How much money do you spend per month total? ___________

12. What is your main source of money? Please select one.
   ☐ mother ☐ father ☐ girlfriend/boyfriend
   ☐ sister /brother ☐ other ☐ work
   ☐ scholarship

13. What do you spend it on? Please select the top three expenses.
   ☐ clothes

Surveying date:
School ID:
Classroom ID:
Student ID:

---

 personal hygiene
 make up
 mobile phone
 sanitary pads
 hair
 school products
 food
 transport
 other

14. How much do you spend on sanitary pads per month? ___________________________

15. Did you miss school in the past 7 days?
 YES NO

If Yes, what was the reason? ____
